# Bile Canalicular Bitter Taste Receptors Inhibit β-Adrenergic Receptor-Induced Lipolysis in Steatotic Hepatocytes

**DOI:** 10.3390/ijms27073226

**Published:** 2026-04-02

**Authors:** Yan-Bo Xue, Shi-Meng Gong, Yuan-Yuan Peng, Defu Yu, Ruhong Zhou, Liquan Huang

**Affiliations:** 1 College of Life Sciences, Zhejiang University, Hangzhou 310058, China; 11907055@zju.edu.cn (Y.-B.X.); 22007052@zju.edu.cn (S.-M.G.); 18089551893@163.com (Y.-Y.P.); 12207087@zju.edu.cn (D.Y.); 2 Shanghai Institute for Advanced Study, Zhejiang University, Shanghai 201203, China

**Keywords:** type 2 taste receptor, βAR, GPCR, bitter compounds, ligand-dependent G protein coupling, liver, bile canaliculus, lipid droplets, cAMP, hepatic steatosis

## Abstract

Bitter taste receptors (TAS2Rs) are G protein-coupled receptors best known for detecting bitter compounds in the oral cavity. However, their expression patterns and physiological roles in the liver remain largely unexplored. Here, we employed molecular and immunohistochemical approaches to demonstrate that multiple TAS2Rs are expressed in human Hep3B cells and mouse primary hepatocytes (MPHs) and co-localized with β-adrenergic receptors (βARs) at the bile canaliculi. Bioluminescence resonance energy transfer (BRET), cAMP assays, and Western blot analyses revealed that certain TAS2Rs exhibit ligand-dependent coupling preferences for the G protein subunits Gαi1, Gαi2, and Gαi3. This coupling leads to inhibition of cAMP production and a reduction in protein kinase A (PKA) substrate phosphorylation. Biochemical assays further showed that TAS2R activation significantly attenuates βAR-mediated lipolysis, as well as the production of glycerol and free fatty acid in both Hep3B cells and MPHs. These effects were partially reversed by small interfering RNA (siRNA)-mediated knockdown of TAS2Rs. Moreover, studies using a steatotic mouse model demonstrated that bitter compounds inhibit lipid droplet degradation, resulting in hepatic triacylglycerol accumulation. Collectively, these findings reveal a role for TAS2Rs in modulating hepatic lipid metabolism and highlight their potential as therapeutic targets for the prevention and treatment of liver diseases.

## 1. Introduction

Bitter taste receptors (TAS2Rs) have been reported in multiple polarized epithelial cell types such as tuft cells, enteroendocrine cells, goblet cells, ciliated cells, and taste bud cells [1,2]. Hepatocytes are not classical epithelial cells, yet they display polarized features. Adjacent hepatocytes form bile canaliculi that resemble the apical domain of epithelia: these closed luminal structures are encircled by actin filaments and sealed by tight junctions, and they transport bile acids, cholesterol, and xenobiotics via specialized transporters typically restricted to the canalicular (apical) membrane, such as the bile salt export pump (BSEP) [3]. However, the expression and function of G protein-coupled receptors (GPCRs) selectively localized to the bile canaliculi have not been reported.

Originally identified from taste buds in the oral cavity for bitter sensing, TAS2Rs are now recognized to exert diverse functions across various organs. In the gastrointestinal and respiratory tracts, TAS2Rs are expressed in tuft cells and participate in innate immune responses through Gαgust-mediated signaling pathways [1]. In airway smooth muscle cells, TAS2R mediates smooth muscle relaxation through the Gαi1/2/3-mediated signaling pathway [4]. In preadipocytes, TAS2R promotes adipogenesis by phosphorylation of extracellular regulated protein kinases 1/2 (ERK1/2) [5,6]. TAS2Rs are able to couple with a variety of G proteins, but the specific G protein types and downstream signaling pathways in tissues outside the oral cavity still need to be fully explored.

Lipid droplets (LDs) serve as a principal intracellular depot for triacylglycerol (TAG) and are governed by multiple signaling cascades [7]. Among them, β-adrenergic receptor (βAR) signaling is well characterized: ligand engagement activates the canonical cyclic adenosine monophosphate (cAMP)–protein kinase A (PKA) pathway, driving phosphorylation of hormone-sensitive lipase (HSL) and perilipin-1 (PLIN1), as well as the translocation of adipose triglyceride lipase (ATGL), thereby promoting robust lipolysis in adipocytes and hepatocytes [8]. In addition, some GPCRs are known to regulate lipid homeostasis [9]. For example, recent studies have shown that multiple olfactory receptors accelerate free fatty acid oxidation through the cAMP/PKA signaling pathway [10,11]. By contrast, Gαi/o-coupled GPCRs—such as the adenosine A1 receptor and GPR109A—suppress cAMP production and inhibit lipid breakdown [12]. Although TAS2Rs are expressed in multiple organs involved in lipid metabolism, such as the liver, adipose tissue, and skeletal muscle, their role in hepatic lipid metabolism remains unclear.

Despite some evidence indicating the TAS2R expression in human and murine liver, their exact subcellular localization, G protein coupling, downstream signaling pathways, and physiological roles in hepatocytes remain poorly understood [13,14]. Here, we systematically characterize the expression of TAS2Rs and their subcellular localization in human and mouse hepatocytes, explore their coupling preference with Gαi1/2/3, and identify and validate their functional crosstalk with βAR in mediating lipolysis. Our findings not only reveal the biased activation of G proteins by TAS2Rs but also uncover a new role for TAS2Rs in hepatic lipid metabolism, which may help identify new therapeutic targets for metabolic liver diseases.

## 2. Results

### 2.1. The Human Hepatocytes Express TAS2Rs in the Apical Membrane of Bile Canaliculi

Human livers express multiple bitter taste receptors; however, their expression in hepatocytes has not been investigated yet [15]. To address this issue, we reanalyzed five human hepatic cell line RNAseq datasets from the GEO database, including: Hep3B (GSE148503), HepG2 (GSE244762), Huh7 (GSE272166), HepaRG (GSE101575) and THLE3 (GSE280026). Our results showed that, among these five cell lines, Hep3B expressed the highest level of TAS2R genes (Figure S1A,B).

**Figure 1:**
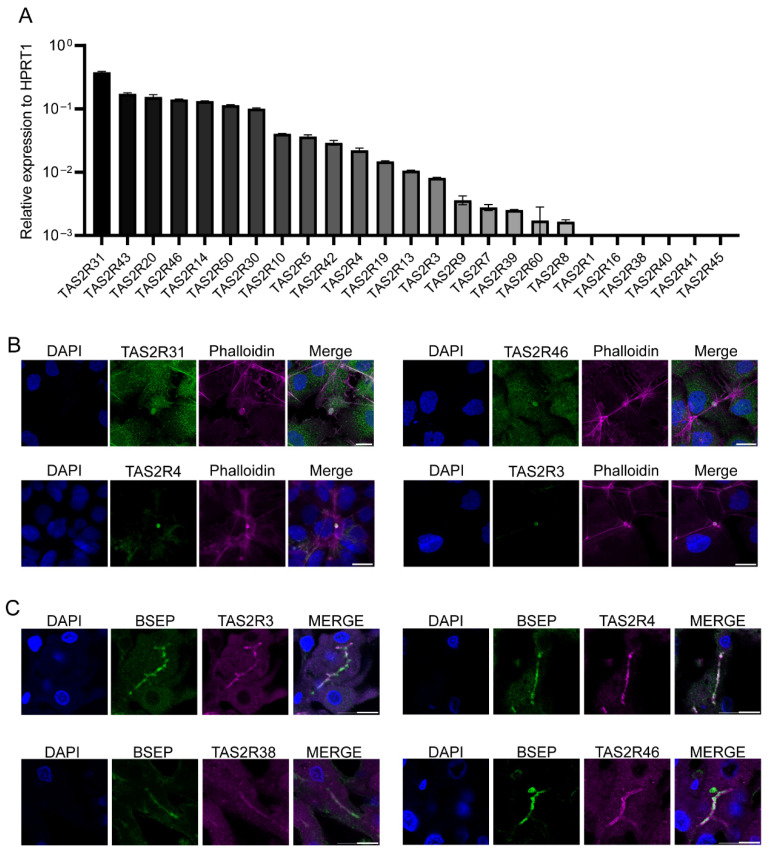
Expression and subcellular localization of TAS2Rs in human hepatocytes. (**A**) Gene expression profiling was performed for all 25 human TAS2R genes in Hep3B cells. Expression levels were calculated as relative ratios between *TAS2Rs* and the internal control gene *HPRT1*. (**B**) Representative immunofluorescence (IF) images of Hep3B cells stained for TAS2R31, TAS2R46, TAS2R4, and TAS2R3 receptors. Phalloidin was used to label the actin-rich pseudo-canaliculi formed by Hep3B cells. (**C**) Representative IF images of human liver sections stained with antibodies against TAS2R3, TAS2R4, TAS2R38, and TAS2R46. BSEP was used as a marker for bile canaliculi in hepatocytes. DAPI was used for nuclear counterstaining. Scale bar: 20 μm.

To confirm the TAS2R expression pattern in Hep3B cells, we carried out qRT-PCR using previously published qPCR primers with additional optimization [16]. The quantitative analysis of the qRT-PCR results indicated that seven TAS2R genes, i.e., *TAS2R31*, *TAS2R43*, *TAS2R20*, *TAS2R46*, *TAS2R14*, *TAS2R50*, and *TAS2R30*, were highly expressed in Hep3B cells, while six other TAS2R genes, *TAS2R10*, *TAS2R5*, *TAS2R42*, *TAS2R4*, *TAS2R19*, and *TAS2R13*, were moderately expressed, and an additional six, i.e., *TAS2R3*, *TAS2R9*, *TAS2R7*, *TAS2R39*, *TAS2R60*, and *TAS2R8*, were expressed at low levels (Figure 1A). The expression of the remaining six *TAS2R* genes, i.e., *TAS2R1*, *TAS2R16*, *TAS2R38*, *TAS2R40*, *TAS2R41*, and *TAS2R45*, was undetectable.

As for the newly identified 26th *TAS2R* gene, *TAS2R2*, it exists as a functional gene in some populations but as a pseudogene in others due to a two-nucleotide deletion (*rs10649477*) caused by a premature stop codon [17]. Our sequencing results of the gene-specific PCR products showed that Hep3B cells carry the homozygous pseudogene version of *TAS2R2* (Figure S2A,B) and are therefore unable to produce a functional receptor protein.

To validate TAS2R expression at the protein level and determine their subcellular localization, we performed immunofluorescence (IF) staining with antibodies against TAS2Rs at different expression levels: highly expressed (TAS2R31 and TAS2R46), moderately expressed (TAS2R4), lowly expressed (TAS2R3) and non-expressed (TAS2R38). The results (Figure 1B) showed that the IF signals for TAS2R31, TAS2R46, TAS2R4 and TAS2R3 were all detected in Hep3B cells, whereas no IF signal was detected for the negative control TAS2R38 or for the negative technical control without the primary antibody (Figure S3). In addition, we observed that these detectable IF signals were predominantly localized to specialized pseudo-canalicular structures.

Furthermore, TAS2Rs have been implicated to interact with β-adrenergic receptors (βAR) [18]. To test this idea, we conducted IF staining against βARs. The results showed that both β1- and β2-adrenergic receptors (β1AR and β2AR) were greatly enriched in the same subcellular structure as the TAS2Rs identified above, i.e., the pseudo-canaliculi (Figure S3).

To assess whether the pseudo-canalicular structure formed by Hep3B cells was functional, we performed live-cell imaging with 5(6)-carboxy-fluorescein diacetate (CFDA) to evaluate directional transport. CFDA would be hydrolyzed into a green fluorescent compound in the cytosol and then transported into the pseudo-canalicular lumen. Green fluorescence in the canaliculi was perceivable 6 min after CFDA loading and increased progressively until 29 min post-loading (Figure S4), indicating the formation of functional pseudo-canalicular similar to what has been observed in HepG2.

Having identified the expression of TAS2Rs in Hep3B cells, we next investigated their expression in human liver tissue. By IF staining, we found that TAS2R3, TAS2R4, TAS2R38, and TAS2R46 were expressed in human liver tissue (Figure 1C). The IF signals of these TAS2Rs were enriched in the cell bodies, forming tubular structures reaching to the adjacent cells. Given their characteristic bile canaliculi localization, we performed double immunostaining with the bile salt export pump (BSEP), a bile canaliculi marker protein, and observed co-localization of all TAS2Rs with BSEP (Figure 1C).

### 2.2. Optimization of Working Concentrations of Bitter Compounds with Minimal Cytotoxicity on Hep3B Cells

TAS2R10, TAS2R14 and TAS2R46 are known to be broadly tuned, while some others are narrowly tuned, and yet the other two, TAS2R19 and TAS2R60, are still orphan receptors, without any known ligands [19]. To activate the TAS2Rs expressed in Hep3B cells with representative ligands, we selected a panel of eight bitter compounds from the database BitterDB [20], including four water-soluble chemicals, aristolochic acid (AA), cromolyn (CRO), denatonium benzoate (DB) and quinine (Q), and four dimethyl sulfoxide (DMSO)-soluble chemicals, aloin (ALO), diphenidol (DIP), hydrocortisone (HC) and saccharin (SAC), some of which, e.g., DIP, Q and DB, are known to activate multiple TAS2Rs, while others, e.g., HC and SAC, activate few TAS2Rs (Table 1).

Among them, DIP, Q and DB can each stimulate 11, eight and seven different TAS2Rs, respectively, while HC, SAC, ALO, AA and CRO can activate one, two, two, three, and three different TAS2Rs found in Hep3B cells, respectively. Since many bitter-tasting compounds are somewhat cytotoxic, to determine the concentrations that cause no or minimal toxic effects on Hep3B cells, we performed cell viability assays on Hep3B cells with these bitter compounds at various concentrations for 24 h (Figure S5).

Results showed that up to 800 μM, CRO had no significant effect on the Hep3B cell viability (Figure S5B), while DB, ALO, HC and SAC did exert some adverse effect only after their concentrations reached 800, 200, 400 and 5000 μM, respectively (Figure S5C,E,G,H). Since the known effective stimulating concentrations on the corresponding TAS2Rs are well below the respective concentrations that decrease the cell viability, we decided to use 100 μM as the working concentration in the subsequent experiments for CRO, DB, ALO and HC and 2000 μM for SAC, which can activate TAS2Rs without any negative effect on Hep3B cells. On the other hand, AA, Q and DIP exhibited some negative effects on Hep3B cell viability starting from 6, 25 and 6 μM, respectively (Figure S5A,D,F). Based on previous studies, we selected 25 µM for AA and Q and 100 µM for DIP as the working concentrations to ensure the activation of the corresponding TAS2Rs with minimal cell viability reduction [21,22,23].

Since DMSO was used as a solvent for some bitter compounds, we also tested its effect on Hep3B viability. The results (Figure S5I) indicated that up to 2% (*v*/*v*), DMSO had no significant effect on Hep3B cell viability. In this study, no more than 0.8% DMSO was used in the working solutions to avoid any adverse effect.

### 2.3. TAS2Rs Activate Gαi1/2/3 Subtypes in a Ligand-Dependent Manner

While they are primarily coupled with Gαgust in taste buds, TAS2Rs in many extra-oral tissues have been found to mediate downstream signaling pathways via Gαi1/2/3 [24]. However, the specific G protein subtypes involved vary across different studies. Therefore, we next investigated the coupling relationships between TAS2Rs expressed in Hep3B cells and Gαi1, Gαi2, and Gαi3.

To systematically study G protein coupling, we employed the bioluminescence resonance energy transfer (BRET)-based TRUPATH system, which allows sensitive detection of intracellular G protein activation [25]. HEK293T cells were co-transfected with four components—TAS2R46, Gα-RLuc8, Gβ3, and Gγ9-GFP2—using Gαi1, Gαi2, or Gαi3 as the Gα subunit. Because GPCR activation induces the dissociation of the heterotrimeric G proteins, receptor-mediated G protein activation is reflected by a decrease in the BRET ratio.

**Figure 2:**
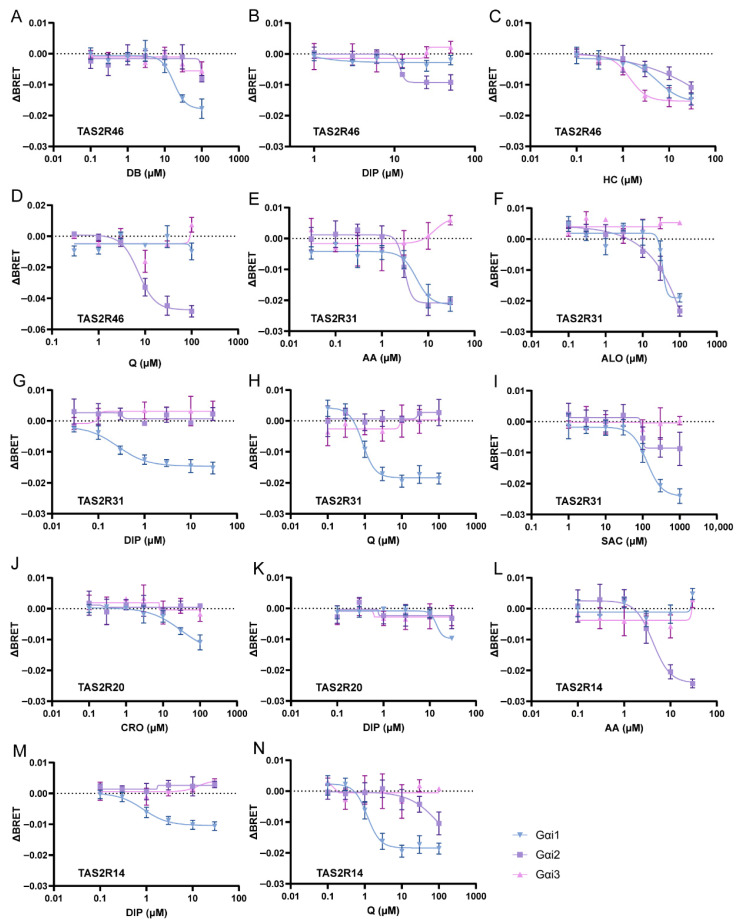
BRET assays of TAS2R coupling to the G proteins Gαi1, Gαi2 and Gαi3 in response to different bitter compounds. (**A**–**D**) Dose–response curves of Gαi1-Gβγ, Gαi2-Gβγ or Gαi3-Gβγ dissociation in TAS2R46-overexpressing HEK293T cells in response to DB (**A**), DIP (**B**), HC (**C**) and Q (**D**). (**E**–**I**) Dose–response curves of Gαi1-Gβγ, Gαi2-Gβγ or Gαi3-Gβγ dissociation in TAS2R31-overexpressing HEK293T cells in response to AA (**E**), ALO (**F**), DIP (**G**), Q (**H**) and SAC (**I**). (**J**,**K**) Dose–response curves of Gαi1-Gβγ, Gαi2-Gβγ or Gαi3-Gβγ dissociation in TAS2R20-overexpressing HEK293T cells in response to CRO (**J**) and DIP (**K**). (**L**–**N**) Dose–response curves of Gαi1-Gβγ, Gαi2-Gβγ or Gαi3-Gβγ dissociation in TAS2R14-overexpressing HEK293T cells in response to AA (**L**), DIP (**M**) and Q (**N**). Values are represented as mean ± s.e.m. of 3 independent experiments (n = 3). AA, aristolochic acid; ALO, aloin; CRO, cromolyn; DB, denatonium benzoate; DIP, diphenidol; HC, hydrocortisone; Q, quinine; SAC, saccharin.

As shown in Figure 2A–D, activation of TAS2R46 by bitter compounds activated Gαi1, Gαi2, and Gαi3 in a dose-dependent manner. However, individual ligands exhibited distinct coupling preferences. DB selectively activated only Gαi1 (EC_50_ = 17.47 μM), whereas DIP and Q selectively activated only Gαi2 (EC_50_ = 11.76 μM and 7.18 μM, respectively). HC, however, activated all three Gαi subunits: TAS2R46 showed the highest sensitivity to HC when coupled with Gαi3 (EC_50_ = 1.41 μM), whereas coupling with Gαi1 resulted in a lower sensitivity (EC_50_ = 5.48 μM). When coupled with Gαi2, the EC_50_ value could not be accurately determined due to incomplete coverage of the activation range, although sensitivity was clearly reduced.

To determine whether this ligand-dependent coupling bias is a general feature of bitter taste receptors, we next examined additional TAS2Rs. For TAS2R31, coupling was observed with Gαi1 and Gαi2, but not with Gαi3 (Figure 2E–I). Among the five ligands tested, AA, ALO, and SAC activated both Gαi1 and Gαi2, although TAS2R31 exhibited lower sensitivity to AA when coupled with Gαi1 (EC_50_ = 5.53 μM) compared with Gαi2 (EC_50_ = 3.05 μM). Similar trends were observed for ALO and SAC. In contrast, DIP and Q selectively activated Gαi1 but failed to activate Gαi2 or Gαi3.

For TAS2R20, only Gαi1 activation was detected in response to CRO and DIP (Figure 2J,K). CRO exhibited an EC_50_ value of 30.87 μM, whereas the EC_50_ for DIP could not be accurately determined. No activation of Gαi2 or Gαi3 was observed.

TAS2R14 activated both Gαi1 and Gαi2, while none of the tested ligands induced Gαi3 activation (Figure 2 L–N). Q activated both Gαi1 (EC_50_ = 1.17 μM) and Gαi2, although the EC_50_ for Gαi2 could not be reliably calculated. AA selectively activated Gαi2 (EC_50_ = 4.17 μM), whereas DIP selectively activated Gαi1 (EC_50_ = 0.85 μM).

Collectively, these results demonstrate that bitter taste receptors can activate multiple Gαi subtypes, but the specific coupling pattern depends on the ligands used, indicating a pronounced ligand-biased G protein activation.

### 2.4. TAS2Rs Inhibit cAMP/PKA Signaling Pathways in Hep3B Cells

**Figure 3:**
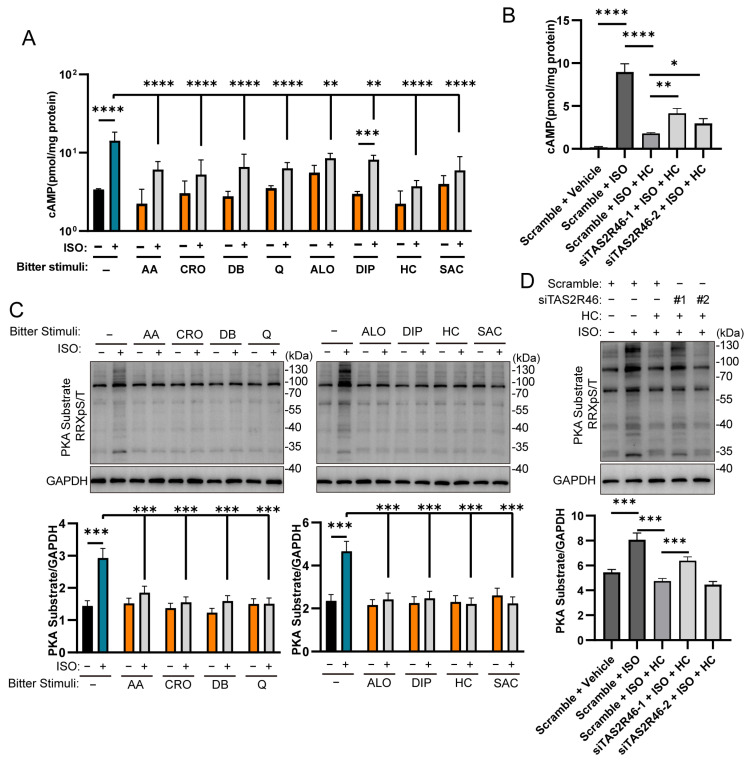
Effect of TAS2R activation by bitter compounds on isoproterenol-induced cAMP production and PKA substrate phosphorylation in steatotic Hep3B cells with or without TAS2R knockdown. (**A**) Intracellular cAMP levels in Hep3B cells treated with ISO (50 μM) and one of the eight bitter stimuli (25 μM AA, 100 μM CRO, 100 μM DB, 25 μM Q, 100 μM ALO, 100 μM DIP, 100 μM HC, or 2 mM SAC) alone or in combination were measured. (**B**) cAMP levels in TAS2R46 siRNA-transfected Hep3B cells treated with ISO (50 μM) and HC (100 μM) alone or in combination were assessed. (**C**) Western blot was used to detect PKA substrate phosphorylation in Hep3B cells treated with ISO (50 μM) and one of the eight bitter stimuli (25 μM AA, 100 μM CRO, 100 μM DB, 25 μM Q, 100 μM ALO, 100 μM DIP, 100 μM HC, or 2 mM SAC) alone or in combination. (**D**) Western blot was used to detect PKA substrate phosphorylation in TAS2R46 siRNA-transfected Hep3B cells treated with ISO (50 μM) and HC (100 μM) alone or in combination. n = 3; *: *p* < 0.05; **: *p* < 0.01; ***: *p* < 0.001; ****: *p* < 0.0001. AA, aristolochic acid; ALO, aloin; cAMP, cyclic adenosine monophosphate; CRO, cromolyn; DB, denatonium benzoate; DIP, diphenidol; HC, hydrocortisone; ISO, isoproterenol; PKA, protein kinase A; Q, quinine; SAC, saccharin.

Gαi was named for its ability to inhibit adenylate cyclase (AC) activity, reducing cAMP synthesis. Since TAS2Rs are coupled to Gαi1/2/3, we next performed ELISA assays on Hep3B cells to determine the cAMP levels. In Hep3B cells, activation of βARs with their specific agonist isoproterenol (ISO) at 50 μM for 24 h significantly increased intracellular cAMP levels compared to untreated controls (Figure 3A). However, co-treatment of ISO with any of the eight bitter compounds led to a significant reduction in cAMP concentrations. The reduction caused by seven of the eight bitter compounds, i.e., AA, CRO, DB, Q, ALO, HC and SAC, was nearly complete, resulting in the intracellular cAMP levels being statistically indifferent from those of the cells stimulated by the corresponding bitter compounds alone. In contrast, the reduction engendered by DIP was partial since the cAMP levels of the cells treated with ISO plus DIP were significantly higher than those of the cells treated with DIP alone.

To verify whether the inhibition of cAMP elevation by bitter compounds is mediated by TAS2Rs, we carried out gene knockdown experiments using small interfering RNAs (siRNAs) to downregulate the target TAS2Rs. Given that some bitter compounds activate multiple TAS2Rs, we selected *TAS2R46*, which is the sole TAS2R that can be activated by HC in Hep3B cells (Table 1). Three siRNAs were designed for the target gene.

To test the efficacy of these siRNAs, we transfected the siRNAs into human Hep3B cells and performed qRT-PCR. The results showed that two of the siRNAs, siTAS2R46-1 and siTAS2R46-2, significantly reduced the *TAS2R46* expression in Hep3B cells to 50% and 40% of that of the scramble, respectively, while the third one, siTAS2R46-3, did not (Figure S6A). IF staining confirmed the reduced TAS2R46 protein levels following the transfection of siTAS2R46-1 and siTAS2R46-2 but not siTAS2R46-3 (Figure S6B,C). Thus, in the subsequent experiments, only siTAS2R46-1 and siTAS2R46-2 were used.

Next, we performed a similar cAMP assay on Hep3B cells transfected with siRNAs to verify the role of TAS2Rs in inhibiting cAMP production. In the scramble-transfected Hep3B cells, while ISO still elevated the cAMP level, co-treatment with HC decreased it significantly, confirming that transfection itself did not affect receptor function (Figure 3B). However, knockdown of TAS2R in Hep3B cells (siTAS2R46-1 or -2-transfected) significantly impaired the bitter compound-mediated reduction in the cAMP levels, demonstrating that TAS2R activation is required for bitter-compound-inhibiting cAMP signaling.

cAMP is known to regulate the activity of protein kinase A (PKA), which phosphorylates a number of substrate proteins, including HSL and PLN1 that regulates LD degradation [8]. To evaluate how bitter-tasting compounds affect PKA phosphorylation, we performed Western blot analysis with an antibody against the PKA-phosphorylated substrate RRXpS/T. In the Hep3B cells treated with ISO alone, the phosphorylation of PKA substrates was significantly upregulated compared with the corresponding untreated cells (Figure 3C). The co-treatment of ISO with one of the eight bitter compounds, i.e., AA, CRO, DB, Q, ALO, DIP, HC, and SAC, abolished the upregulated phosphorylation. siRNA-transfected Hep3B cells showed that in the scramble-transfected Hep3B cells (Figure 3D), ISO significantly upregulated PKA substrate phosphorylation, which was inhibited by the bitter compound HC, confirming that the functions of βARs and TAS2Rs were not altered by siRNA transfection per se. The transfection of siTAS2R46-1 but not siTAS2R46-2 partially eliminated HC-evoked downregulation of PKA substrate phosphorylation (Figure 3D).

These results indicate that activation of TAS2Rs in Hep3B cells inhibits the cAMP/PKA signaling pathway.

### 2.5. TAS2Rs Inhibit βARs-Mediated Lipolysis in Hep3B Cells

Approximately 50 different GPCRs have been reported in the liver, many of which play roles in the progression of metabolic dysfunction-associated fatty liver disease. Among them, several olfactory receptors and β-adrenergic receptors (βARs) were found to participate in triglyceride metabolism in hepatocytes by regulating the cAMP/PKA signaling pathway [26]. Since lipid droplets (LDs) are mainly used for triacylglycerol (TAG) storage in cells, we performed LD analysis to assess the roles of TAS2Rs and βARs in TAG metabolism in Hep3B cells.

**Figure 4:**
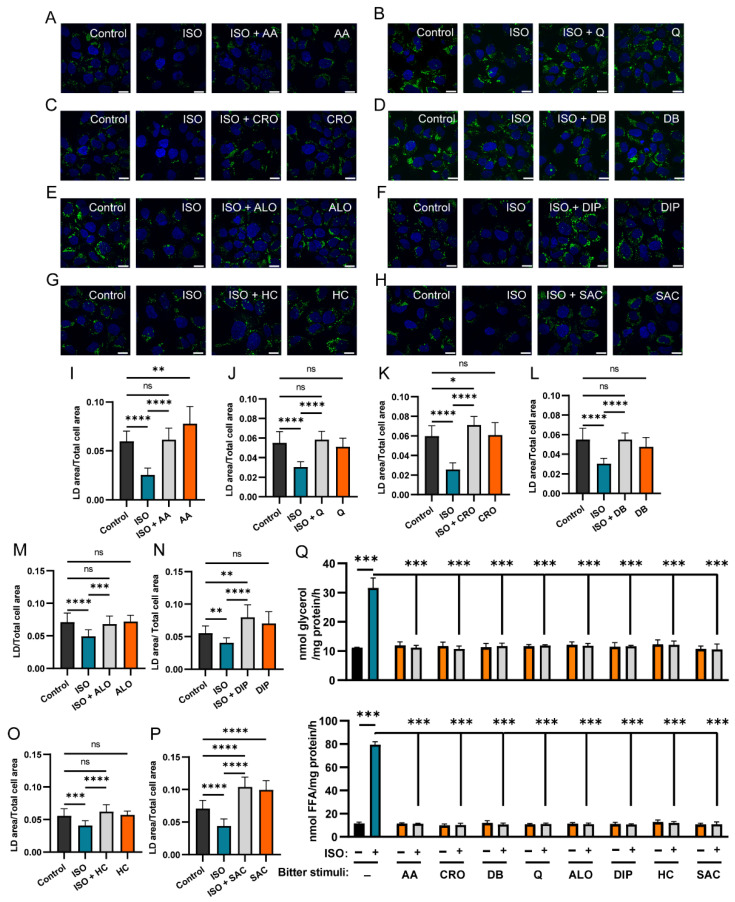
Assessment of lipid droplet degradation and lipolysis in steatotic Hep3B cells co-treated with ISO and bitter stimuli. (**A**–**H**) Representative images showing green LD signal in Hep3B cells treated for 24 h with the following compounds: 50 μM ISO, 50 μM ISO plus 25 μM AA, or 25 μM AA alone (**A**); 50 μM ISO, 50 μM ISO plus 25 μM Q or 25 μM Q alone (**B**); 50 μM ISO, 50 μM ISO plus 100 μM CRO or 100 μM CRO alone (**C**); 50 μM ISO, 50 μM ISO plus 100 μM DB or 100 μM DB alone (**D**); 50 μM ISO, 50 μM ISO plus 100 μM ALO or 100 μM ALO alone (**E**); 50 μM ISO, 50 μM ISO plus 100 μM DIP or 100 μM DIP alone (**F**); 50 μM ISO, 50 μM ISO plus 100 μM HC or 100 μM HC alone (**G**); 50 μM ISO, 50 μM ISO plus 2 mM SAC or 2 mM SAC alone (**H**); control cells were treated with the solvent for each bitter compound. DAPI was used for nuclear staining. (**I**–**P**) Results in (**A**–**H**) were quantified by LD areas and normalized against the cell areas (n > 10). (**Q**) Glycerol and FFA production were measured in Hep3B cells treated with ISO (50 μM) and one of the eight bitter stimuli (25 μM AA, 100 μM CRO, 100 μM DB, 25 μM Q, 100 μM ALO, 100 μM DIP, 100 μM HC, or 2 mM SAC) alone or in combination (n = 3). ns: no statistical significance; *: *p* < 0.05; **: *p* < 0.01; ***: *p* < 0.001; ****: *p* < 0.0001. AA, aristolochic acid; ALO, aloin; CRO, cromolyn; DB, denatonium benzoate; DIP, diphenidol; HC, hydrocortisone; Q, quinine; SAC, saccharin; ISO, isoproterenol. Scale bar: 20 μm.

Our results indicated that in oleic acid pre-loaded Hep3B cells, ISO treatment significantly reduced LD contents compared with that of the control (Figure 4A–P). We then tested the effect of eight bitter compounds listed in Table 1 on the βARs-induced lipolysis in Hep3B cells. The results indicated that combining ISO with any one of the eight bitter compounds significantly increased LD contents compared to ISO alone, indicating that these compounds inhibit βAR-mediated lipolysis (Figure 4A–P).

The inhibitory potencies varied among the eight compounds. AA, Q, DB, ALO and HC completely abolished ISO-induced lipolysis, restoring the LD content to levels comparable to the control (Figure 4A,B,D,E,G,I,J,L,M,O). In contrast, CRO, DIP and SAC resulted in even higher LD contents than the control (Figure 4C,F,H,K,N,P). AA and SAC alone did significantly increase LD contents (Figure 4A,H,I,P), while the other six compounds did not (Figure 4B–G,J–O).

The variations suggest that some of these bitter compounds may also act on other receptors, ion channels or proteins in addition to TAS2Rs in Hep3B cells. One possibility was that DB might function as an α adrenergic receptor (αAR) antagonist [27]. However, replacing DB with a specific αAR antagonist, phentolamine, did not inhibit ISO-induced lipolysis, thus excluding the possibility that DB acts via αAR antagonism (Figure S7).

During lipolysis, TAG, the primary component of LD, is hydrolyzed into glycerol and free fatty acids (FFAs) [8]. To determine whether ISO and bitter compounds are involved in the regulation of this hydrolysis, we measured the glycerol and FFA levels in the culture medium of Hep3B cells in response to these stimuli. In these cells, ISO significantly increased the production of both glycerol and FFA (Figure 4Q), indicating enhanced lipolytic activity. Co-treatment of Hep3B cells with ISO plus one of the eight bitter compounds, AA, CRO, DB, Q, ALO, DIP, HC and SAC, abolished the ISO-induced increase in glycerol and FFA production, which remained at the same levels of the unstimulated control cells. Notably, none of the bitter-tasting compounds alone significantly affected glycerol or FFA production.

**Figure 5:**
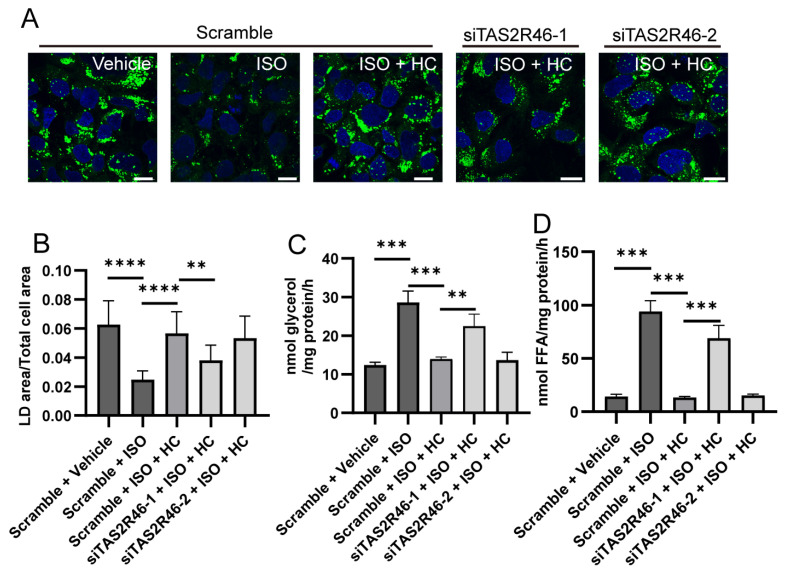
Effect of TAS2R46 knockdown on hydrocortisone (HC)-mediated changes in lipolysis in steatotic Hep3B cells. (**A**) Representative images showing green lipid droplet (LD) signals in siRNA-transfected Hep3B cells after 24 h treatment with: vehicle, 50 μM isoproterenol (ISO), or 50 μM ISO plus 100 μM HC. (**B**) Quantification of LD areas from panel (**A**), normalized against the respective cell areas (n > 10). (**C**,**D**) Glycerol (**C**) and free fatty acid (FFA) (**D**) production was measured in TAS2R46 siRNA-transfected Hep3B cells treated with ISO (50 μM) and HC (100 μM) alone or in combination. ns: no statistical significance; **: *p* < 0.01; ***: *p* < 0.001; ****: *p* < 0.0001. Scale bar: 20 μm.

We then performed LD analysis following TAS2R knockdown (Figure 5A,B). Scramble siRNA transfection did not alter ISO-induced LD reduction or HC-mediated inhibition, as seen in the un-transfected Hep3B cells. However, HC-mediated inhibition on LD reduction remained only in the siTAS2R46-2- but not siTAS2R46-1-transfected Hep3B cells, indicating that siTAS2R46-1-mediated TAS2R46 knockdown impaired its counteractive role against βAR signaling.

We also measured the products of lipolysis. In the scramble-transfected cells, the ISO-induced increases in the glycerol (Figure 5C) and FFA (Figure 5D) production and bitter-compound-mediated inhibition on the production remained intact, confirming that siRNA transfection per se did not affect the function of βARs or TAS2Rs. In siTAS2R46-1- but not siTAS2R46-2-transfected Hep3B cells, HC failed to suppress the production of glycerol or FFA as much as in the scramble-transfected cells, indicating that TAS2R46 was required for the HC-mediated inhibition.

These results demonstrate that activation of TAS2Rs in Hep3B inhibits LD degradation and lipolysis.

### 2.6. Tas2r Expression Profiling in Mouse Primary Hepatocytes

Previous studies reported discrepancies in the expression profiles of Tas2rs in mouse hepatocytes [13,14,28]. Even less was known about their functional roles in these cells or in the liver. We first isolated mouse primary hepatocytes (MPHs) and performed qRT-PCR to validate *Tas2r* expression. Using the optimized qPCR primers, we detected eight *Tas2rs* (Figure S8A), among which *Tas2r138* and *Tas2r126* were highly expressed, *Tas2r137*, *Tas2r135*, and *Tas2r143* were moderately expressed, and *Tas2r113*, *Tas2r108* and *Tas2r140* were lowly expressed. The remaining 27 Tas2rs were not detected.

Our results included six Tas2rs previously reported by others [13]. However, we did not detect *Tas2r109* or *Tas2r130*, contrary to a previous study [14]. RT-PCR confirmed the absence of the amplified products from these hepatocytes, while the positive control reactions on the adipose tissue generated the expected bands (Figure S8B,C). This result indicates that *Tas2r109* and *Tas2r130* may be expressed in non-parenchymal cells. We also found that *Tas2r144* was not expressed in hepatocytes, although the PCR product of a partial sequence initially suggested otherwise (Figure S8B). The full coding sequence of *Tas2r144* was amplified only from the adipose tissue, not from the hepatocytes (Figure S8D).

### 2.7. Tas2rs Inhibited cAMP/PKA Signaling Pathways in MPHs

We analyzed the activation profiles of the Tas2rs expressed in MPHs by the eight bitter substances used in the previous experiments. Four of them (ALO, AA, CRO, and HC) did not activate any of these Tas2rs in MPHs, while the other four (DB, DIP, Q, and SAC) activated one or more of the Tas2rs expressed in MPHs. The specific minimum activation concentrations are summarized in Table 2. Among them, the bitter stimuli DB (100 μM) and SAC (2000 μM) each activated only one Tas2r, Tas2r135, while DIP activated two Tas2rs, and Q activated four Tas2rs. Given that our results in Hep3B indicate TAS2R-mediated inhibition on the cAMP/PKA signaling pathway, we next also examined the effect of Tas2rs on the cAMP/PKA signaling pathway in MPHs cultured in a collagen sandwich.

**Figure 6:**
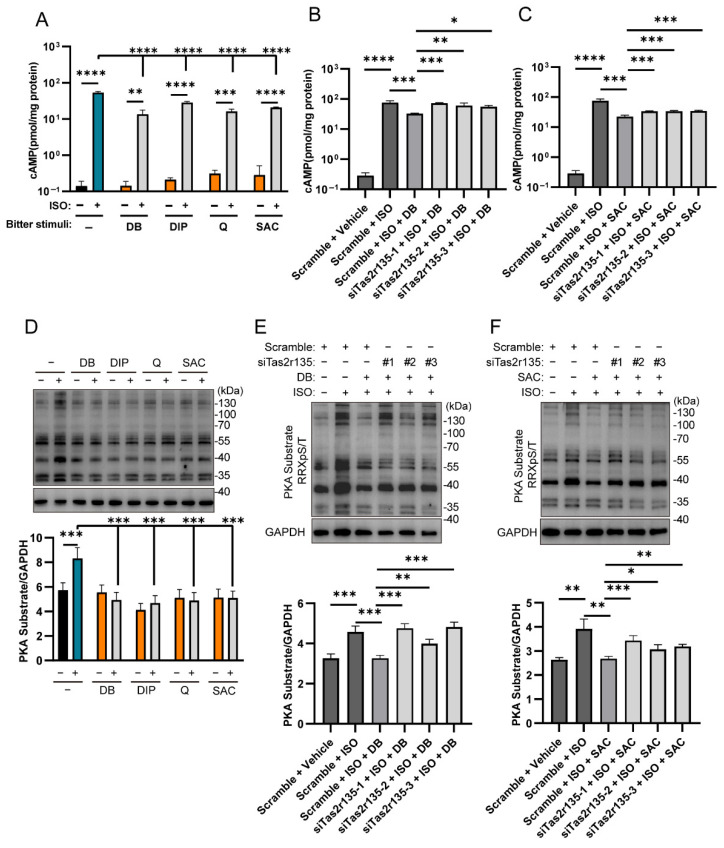
Effects of Tas2r activation by bitter compounds on isoproterenol (ISO)-induced changes in cAMP levels and PKA substrate phosphorylation in mouse primary hepatocytes (MPHs) with or without Tas2r knockdown. (**A**) Intracellular cAMP levels in MPHs treated with ISO (50 μM) and one of the four bitter stimuli (100 μM DB, 100 μM DIP, 25 μM Q, or 2 mM SAC) alone or in combination were measured. (**B**,**C**) cAMP levels in Tas2r135 siRNA-transfected MPHs treated with ISO (50 μM) and DB (100 μM) (B) or SAC (2 mM) (**C**) alone or in combination were assessed. (**D**) Western blot was used to detect PKA substrate phosphorylation in Hep3B cells treated with ISO (50 μM) and one of the four bitter stimuli (100 μM DB, 100 μM DIP, 25 μM Q, or 2 mM SAC) alone or in combination. (**E**) Western blot was used to detect PKA substrate phosphorylation in *Tas2r135* siRNA-transfected MPHs treated with ISO (50 μM) and DB (100 μM) alone or in combination. (**F**) Western blot was used to detect PKA substrate phosphorylation in *Tas2r135* siRNA-transfected MPHs treated with ISO (50 μM) and SAC (2 mM) alone or in combination. n = 3; *: *p* < 0.05; **: *p* < 0.01; ***: *p* < 0.001; ****: *p* < 0.0001. cAMP, cyclic adenosine monophosphate; DB, denatonium benzoate; DIP, diphenidol; PKA, protein kinase A; Q, quinine; SAC, saccharin.

We first examined the changes in cAMP levels in MPHs after treatment with the bitter stimuli and ISO. As shown in Figure 6A, ISO increased the intracellular cAMP levels compared to the untreated control cells. The co-treatment of ISO with DB, DIP, Q, or SAC significantly reduced this elevation but not the cAMP level of the bitter compounds alone, suggesting that there are still some differences between PMH and Hep3B cells.

For the MPH, we chose *Tas2r135*, which is the sole Tas2r that can be activated by SAC (2000 μM) or DB (100 μM) in MPHs (Table 2). Tas2r knockdown experiments were also carried out to determine whether the regulation of lipolysis by bitter compounds was Tas2rs-dependent in the MPH. In the collagen-sandwich-cultured MPH, *Tas2r135*-targeting siRNAs siTas2r135-1, -2 and -3 effectively downregulated the *Tas2r135* mRNA level to approximately 51% of that of the scramble siRNA (Figure S9). Due to the lack of high-quality antibodies against the mouse Tas2r135 receptor, the protein level change in the siRNA-transfected MPH was not assessed.

In the scramble-transfected MPH, ISO still elevated cAMP, while co-treatment with one of the bitter compounds, DB (Figure 6B) or SAC (Figure 6C), decreased cAMP levels significantly, confirming that the functions of βARs and TAS2Rs were not altered in the MPH either. However, knockdown of *Tas2r135* in the MPH (siTas2r135-1, -2, or -3-transfected) significantly impaired the bitter-compound-mediated reduction in cAMP levels (Figure 6B,C), demonstrating that Tas2r activation is required for bitter-compound-inhibiting cAMP signaling.

Next, we examined the changes in PKA substrate phosphorylation levels after treating MPHs with the bitter stimuli (DB, DIP, Q and SAC) and ISO. As shown in Figure 6D, ISO significantly increased PKA substrate phosphorylation levels compared to the untreated control cells. However, when ISO and the bitter stimuli were added simultaneously, PKA substrate phosphorylation levels were significantly reduced compared to the cells treated with ISO alone. There was no significant difference between the co-treatment group with the bitter stimuli and ISO and the group treated with the corresponding bitter stimuli alone.

In the scramble-transfected MPHs, ISO still significantly upregulated PKA substrate phosphorylation, which was inhibited by the bitter compound DB (Figure 6E) or SAC (Figure 6F), confirming that the functions of βARs and TAS2Rs were not altered by siRNA transfection per se. Transfection of siTas2r135-1, -2 or -3 significantly restored ISO-induced PKA substrate phosphorylation in the presence of the bitter compound DB (Figure 6E) or SAC (Figure 6F).

PKA substrates encompass key lipases and their regulatory proteins, notably hormone-sensitive lipase (HSL) and perilipin 1 (PLIN1), which serve as direct molecular switches for lipid droplet (LD) breakdown. To further define the downstream targets of this signaling cascade, we specifically examined HSL phosphorylation. Western blot analysis results showed that ISO treatment robustly induced HSL phosphorylation, which was effectively counteracted by co-treatment with the four bitter stimuli (DB, DIP, Q, and SAC) (Figure S10A). Furthermore, siRNA-mediated knockdown of *Tas2r135* (siTas2r135-1, -2, or -3) significantly diminished the inhibitory effect of DB (Figure S10B) and SAC (Figure S10C) on HSL phosphorylation, whereas the scramble control siRNA had no such impact. These findings collectively confirm that *Tas2r135* is essential for the bitter-compound-mediated suppression of HSL activation.

These results indicate that activation of Tas2rs in MPHs also inhibits the cAMP/PKA signaling pathway.

### 2.8. Tas2rs Inhibit LD Degradation and Lipolysis in MPH

**Figure 7:**
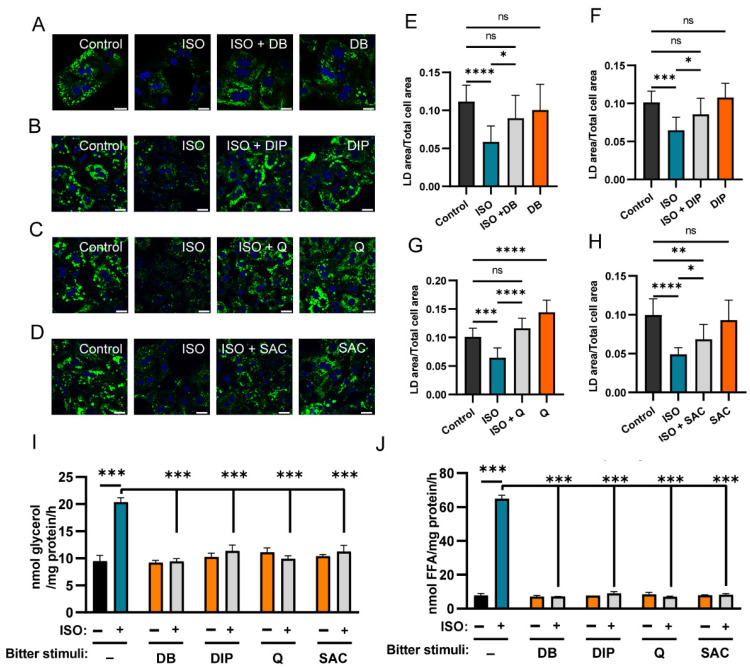
Assessment of lipid droplet degradation and lipolysis in mouse primary hepatocytes (MPH) co-treated with isoproterenol (ISO) and bitter stimuli. (**A**–**D**) Representative images showing green LD signal in MPHs after 24 h treatment with: 50 μM ISO, 50 μM ISO plus 100 μM DB, or 100 μM DB alone (**A**); 50 μM ISO, 50 μM ISO plus 100 μM DIP, or 100 μM DIP alone (**B**); 50 μM ISO, 50 μM ISO plus 25 μM Q, or 25 μM Q alone (**C**); 50 μM ISO, 50 μM ISO plus 2000 μM SAC, or 2000 μM SAC alone (**D**). Control cells were treated with the solvent for each bitter compound. DAPI was used for nuclear staining. Scale bar: 20 μm. (**E**–**H**) Quantitative analyses of LD areas in (**A**–**D**), normalized against the respective cell areas (n > 10). (**I**,**J**) Glycerol (**I**) and FFA (**J**) production was measured in mouse primary hepatocytes treated with ISO (50 μM) and one of the four bitter stimuli (100 μM DB, 100 μM DIP, 25 μM Q, or 2 mM SAC) alone or in combination. ns: no statistical significance; *: *p* < 0.05; **: *p* < 0.01; ***: *p* < 0.001; ****: *p* < 0.0001. DB, denatonium benzoate; DIP, diphenidol; Q, quinine; SAC, saccharin; FFA, free fatty acid; LD, lipid droplet.

Following the verification of cAMP/PKA pathway inhibition by Tas2r activation, we next conducted LD analysis on MPHs cultured in a collagen sandwich. Following 24 h pretreatment with 200 μM oleic acids, ISO significantly reduced LD contents in MPHs (Figure 7A–H and Figure S11), which is consistent with the above Hep3B results. Application of the eight bitter compounds revealed that AA, ALO, CRO, and HC did not inhibit lipolysis (Figure S11), which is in alignment with the absence of their target Tas2rs in MPHs (Table 2). In contrast, DB, DIP, Q, or SAC significantly inhibited LD degradation in MPHs (Figure 7A–H). Interestingly, Q alone increased LD content in MPHs (Figure 7C,G), while DB, DIP or SAC alone did not (Figure 7A,B,D–F,H).

We also tested the production of glycerol and FFA in MPHs. Following ISO treatment, the production of glycerol and FFA significantly increased. But treatment of MPHs with ISO plus one of the four bitter compounds DB, DIP, Q, or SAC inhibited ISO-induced glycerol (Figure 7I) and FFA (Figure 7J) production. And the application of the bitter compounds alone had no effect on lipolysis.

**Figure 8:**
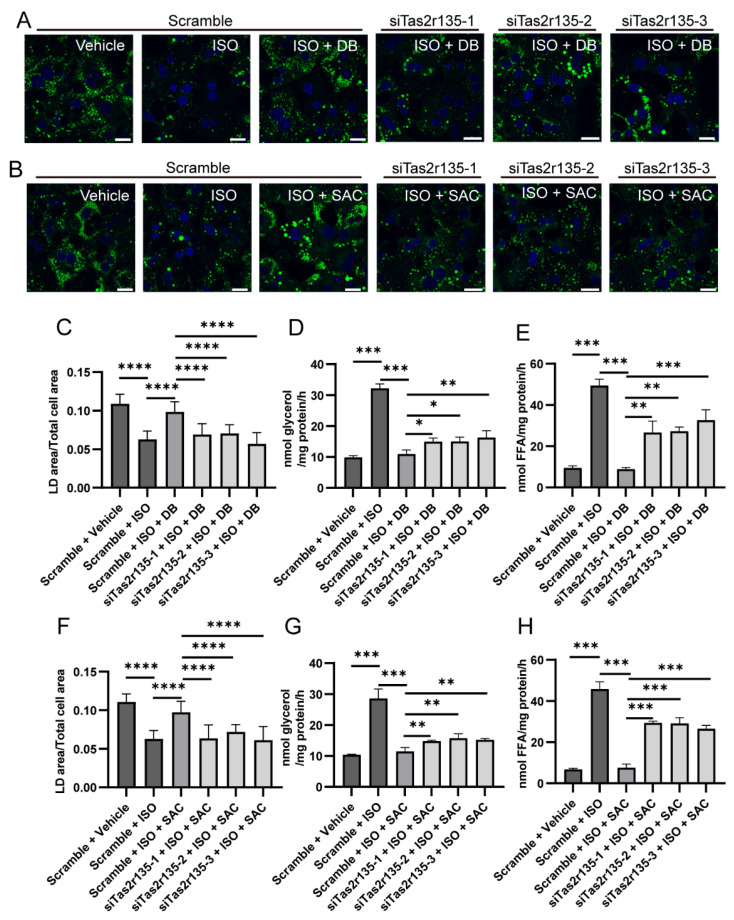
Effect of *Tas2r135* knockdown on denatonium benzoate (DB)- and saccharin (SAC)-mediated changes in lipolysis in mouse primary hepatocytes (MPHs). (**A**,**B**) Representative images showing green LD signals in siRNA-transfected MPHs after 24 h treatment with vehicle, 50 μM ISO, or 50 μM ISO plus 100 μM DB (**A**) or 50 μM ISO plus 2000 μM SAC (**B**). (**C**) Quantification of LD areas from the panels in (**A**), normalized to the respective cell areas (n > 10). (**D**,**E**) Glycerol (**D**) and FFA (**E**) production was measured in *Tas2r135* siRNA-transfected MPHs treated with ISO (50 μM) and DB (100 μM) alone or in combination. (**F**) Quantification of LD areas from panels in (**B**), normalized to the respective cell areas (n > 10). (**G**,**H**) Glycerol (**G**) and FFA (**H**) production was measured in *Tas2r135* siRNA-transfected MPHs treated with ISO (50 μM) and SAC (2 mM) alone or in combination. n > 10; ns: no statistical significance; *: *p* < 0.05; **: *p* < 0.01; ***: *p* < 0.001; ****: *p* < 0.0001. ISO, isoproterenol; FFA, free fatty acid; LD, lipid droplet. Scale bar: 20 μm.

Next, we carried out LD analysis and lipolysis product measurements on MPHs transfected with siRNAs (Figure 8). In the scramble-transfected MPHs, ISO reduced LD contents (Figure 8A–C,F) and increased the lipolysis products glycerol (Figure 8D,G) and FFA (Figure 8E,H), as seen in the un-transfected MPHs (Figure 7), indicating that siRNA transfection per se did not affect βAR activity. Furthermore, the results from the treatment with ISO plus DB (Figure 8A,C–E) or SAC (Figure 8B,F–H) showed the inhibition on lipolysis in the scramble-transfected MPHs, indicating that the Tas2r135 receptor’s function was intact.

However, the effects of DB or SAC were significantly suppressed in the *Tas2r135* knockdown MPHs (siTas2r135-1/-2/-3-transfected) compared to the corresponding scramble-transfected cells. In the siTas2r135-1-, -2- or -3-transfected MPHs, LD analysis revealed that significantly more LDs were retained in the cells (Figure 8A–C,F). Concomitantly, the knockdown of *Tas2r135* partially reversed the inhibition on the glycerol and FFA production incurred by DB (Figure 8D,E) or SAC (Figure 8G,H). Taken together, these results demonstrate that bitter-compound-elicited inhibition on lipolysis is dependent on Tas2r receptor activity.

These results indicate that activation of Tas2rs in MPHs also inhibits LD degradation and lipolysis.

### 2.9. Bitter Stimuli Also Inhibit Lipolysis in Steatotic Mouse Liver

**Figure 9:**
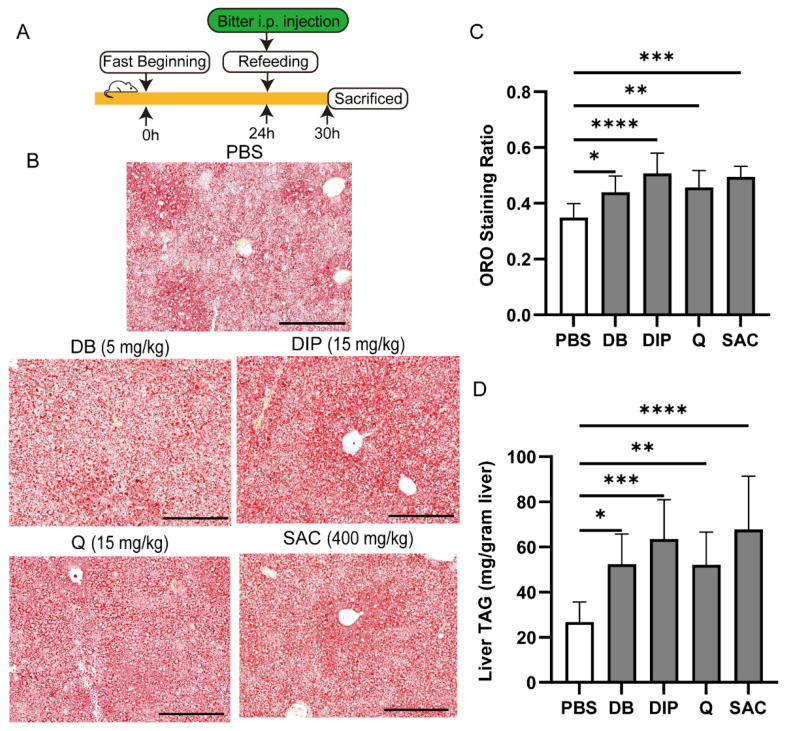
In vivo assessment of bitter stimuli’s inhibition on hepatic lipolysis in a fasting–refeeding mouse model. (**A**) Schematic of experimental procedure: fasting, bitter stimulus or PBS injection, refeeding and tissue collection. (**B**) Representative images of ORO-stained liver sections from mice treated with PBS or bitter compounds following the protocol in (**A**). Scale bar: 200 μm. (**C**) Quantification of the ORO-stained areas in the liver sections from the mice treated with PBS or the indicated bitter stimuli was performed. The area of ORO staining was measured by ImageJ (version 1.54) and expressed as a ratio to the tissue area. (**D**) TAG content was measured in the liver tissues from each treatment group. n = 6; ns: no statistical significance; *: *p* < 0.05; **: *p* < 0.01; ***: *p* < 0.001; ****: *p* < 0.0001. i.p., intraperitoneal injection; DB, denatonium benzoate; DIP, diphenidol; Q, quinine; SAC, saccharin; ORO, oil red O; TAG, triacylglycerol.

Having elucidated the effect of TAS2R activation on lipolysis in cultured cells, we next explored its function in vivo with a widely used hepatic steatosis mouse model [29]. As depicted in Figure 9A, mice were fasted for 24 h, during which triacylglycerol (TAG) was mobilized from adipose tissue and accumulated as LDs in hepatocytes. Refeeding not only stimulates the sympathetic nerve and release of norepinephrine but also initiates the degradation of these LDs in the liver. In our experiments, the bitter compounds DB, DIP, Q and SAC were injected intraperitoneally before refeeding, as each of them activates at least one type of Tas2r expressed in mouse liver (Table 2). Six hours post-injection, the liver tissues were harvested and analyzed by oil red O (ORO) staining. All four bitter compounds increased the LD area in the tissue sections, shown by increased ORO staining ratios, indicating the delay of TAG degradation (Figure 9B,C). And assessment of TAG contents in the liver tissues also showed a similar result to that of ORO staining, i.e., significant increases in TAG contents following the injection of these four bitter stimuli (Figure 9D). These results confirm that Tas2r activation by the bitter compounds can inhibit hepatic lipolysis in vivo.

## 3. Discussion

TAS2Rs were initially identified from taste bud cells. However, recent studies have shown that they are expressed in many extraoral tissues and organs, where they play important and unique roles across different physiological systems [24]. Here, we report the expression of TAS2Rs in human and mouse hepatocytes and their pivotal contributions to hepatic lipid metabolism.

Previous studies showed variations in TAS2R expression profiles in hepatic cells. To address this issue, we set out to reanalyze available RNAseq datasets and perform qRT-PCR to validate TAS2R expression (Figure 1 and Figures S1, S2 and S8). Our results confirmed some highly expressed TAS2Rs in the hepatic cell lines and mouse primary hepatocytes. In addition, we also identified some lowly expressed TAS2Rs. One of the undetected *TAS2R*s in Hep3B is *TAS2R38*, which, however, was detected in human liver sections. *TAS2R38* was only weakly expressed in the Huh 7 and HepaRG cell lines. This inconsistency may be attributed to the variations in gene expression profiles among the hepatocytes from different lobes of the liver, whereas these cell lines were derived from one or a few of these hepatocytes. Another possibility is that *TAS2R38* expression may be lost over the passages of these cell lines. Overall, however, these cell lines share many important features of the hepatocytes and have been used as cellular models for studies on liver functions.

To localize TAS2R receptor proteins to subcellular structures, we used TAS2R antibodies and performed immunostaining. The results showed that TAS2R3, TAS2R4, TAS2R38 and TAS2R46 are mostly enriched in the bile canaliculi formed by Hep3B cells or in the human liver tissue (Figure 1). Furthermore, these receptors are co-expressed with β1AR and β2AR to the special structure of bile canaliculus (Figure S3). This is the first report indicating the localization of GPCRs to this special structure, which may be of functional importance. Many specific transporters on the membrane control the directional transport of substances in and out of the bile canaliculus, while the receptors, including TAS2Rs and βARs, constantly monitor the contents in the structure.

The signaling pathway of TAS2Rs in taste buds are different from those in the liver [24]. In taste buds, TAS2Rs usually couple to Gαgust. However, in other tissues, such as airway smooth muscle, Gαi1/2/3 may also be coupled with TAS2Rs. To determine whether TAS2Rs expressed in the liver also interact with Gαi1/2/3, we used the TRUPATH system and systematically characterized the coupling relationships between TAS2Rs and Gαi1, Gαi2, and Gαi3. The results show that multiple TAS2Rs can activate Gαi1, Gαi2, and Gαi3; however, the coupling between TAS2Rs and G proteins is not static (Figure 2). The specific coupling ability of the Gα subunits depends in part on the bitter compounds used, which is in agreement with previous studies showing that TAS2R14 couples to Gαgust or Gαi2 when activated by flufenamic acid [30] but switches to Gαgust or Gαi1 when activated by compound 28.1 [31]. Together, these findings indicate that bitter taste receptors exhibit pronounced biased signaling, governed by both ligand-specific effects and cellular G protein expression profiles.

Recent advances in structural biology have provided increasing evidence for direct interactions between TAS2Rs and Gαi family members. Cryo-electron microscopy structures of TAS2R14 and TAS2R16 revealed direct coupling with Gαi1 and Gαi2 [30,31,32,33,34]. Furthermore, these studies have revealed multiple binding sites on bitter taste receptors. Intracellular allosteric binding sites can engage a variety of ligands and are even more involved in receptor activation than conventional orthosteric sites. The intracellular binding sites are not only composed of the receptor’s transmembrane helix but also contain the α5 helix of the G protein. Since the α5 helix of G proteins is crucial for the G protein recognition of TAS2Rs, the activation of receptors by ligands may be partially involved in the receptor’s selection of the G proteins, providing a possible molecular mechanism for ligand-dependent G protein coupling preferences observed in the present study. A recent study showed that binding of the M_2_ muscarinic acetylcholine receptor to different ligands can induce more than four distinct states of the ligand receptor–G protein complex [35], each of which exhibits a different capacity to activate G proteins. Further studies are needed to identify the mechanisms underlying the bitter-compound-dependent conformational changes in TAS2Rs and their alterations in G protein coupling preferences at the atomic level.

G proteins are composed of α, β, and γ subunits. Although Gβγ subunits are essential for G protein activation, variations in Gβγ composition primarily influence the magnitude of α-subunit activation in the TRUPATH system [25]. Because the Gβ3/Gγ9 combination produces the strongest BRET signal changes and has been widely used in previous bitter taste receptor studies [30,32], we did not substitute these subunits in the present work. It, however, is possible that a unique combination of Gβ and Gγ subunits may trigger specific downstream signaling pathways.

Gαi is involved in the regulation of the cAMP/PKA signaling pathway. To evaluate how activation of TAS2Rs by bitter compounds regulates the intracellular cAMP production and PKA substrate phosphorylation, we carried out cAMP assays and Western blot analyses. The results show that in Hep3B cells, TAS2R activation significantly decreased intracellular cAMP levels, and the ISO-induced increase in cAMP was almost completely abolished (Figure 3). In contrast, in MPHs, although TAS2R activation also significantly reduced cAMP levels, the stimulatory effect of ISO remained significant (Figure 6). We speculate that this difference may be attributed to variations in the receptor expression in these cells.

TAS2R20, TAS2R31, and TAS2R46, which are expressed in Hep3B cells, are primate-specific TAS2Rs; therefore, their Gαi activation properties may differ from those of murine TAS2Rs. In addition, the βAR expression pattern differs between Hep3B cells and MPHs: the former express both β1AR and β2AR, whereas the latter largely express β2AR only [36]. This distinction is also reflected in cAMP responses, as ISO treatment induced a significantly greater increase in cAMP levels in MPHs compared with that in Hep3B cells. Taken together, the differences in both receptor subtypes and expression levels likely contribute to the varying degrees of TAS2R-mediated modulation of intracellular cAMP levels observed in these two types of cells.

Despite the observed differences in cAMP levels, the changes in PKA substrate phosphorylation following TAS2R activation were generally consistent in both Hep3B cells and MPHs (Figure 3 and Figure 6). This suggests that each cell type may maintain its own dynamic equilibrium within the cAMP/PKA signaling network. Regardless of the differences in the receptor expression profiles, TAS2Rs in both Hep3B and MPH cells appear to coordinate closely with βARs to efficiently modulate the cAMP/PKA signaling pathway.

PKA substrates include lipases and several lipase-regulatory proteins, such as hormone-sensitive lipase (HSL) and perilipin 1 (PLIN1), which directly regulate LD breakdown in cells. Our results indicate that indeed HSL phosphorylation was inhibited by bitter compounds (Figure S10). To determine how PKA substrate phosphorylation regulates lipolysis and LD contents in hepatic cells, we quantitatively analyzed LD areas. Our results demonstrate that both TAS2Rs and βARs can modulate LD degradation in hepatocytes (Figure 4, Figure 5, Figure 7 and Figure S11). However, we also observed unexpected effects for certain bitter compounds, such as AA and SAC. When applied alone, both compounds increased the LD area in Hep3B cells. Previous studies have reported that AA can alter lipid metabolites in the kidney [37]. Given that multiple TAS2Rs are expressed in renal tissues [38], these receptors may similarly participate in the regulation of lipid metabolism. SAC, a commonly used artificial sweetener, is known to bind to the sweet taste receptor subunit TAS1R3 and may influence fatty acid transport [39,40]. These findings suggest that bitter compounds may exert diverse biological effects through multiple receptors and signaling pathways. The exact mechanisms underlying these effects require further investigation.

Bitter taste receptors are capable of binding cholesterol as well as a wide range of cholesterol derivatives [41,42]. Notably, both TAS2R46 and TAS2R14 can be activated by multiple steroid hormones. Among these ligands, hydrocortisone (HC) is a particularly potent activator of TAS2R46, and its circulating concentration can reach levels sufficient to activate this receptor under certain physiological or pathological conditions. Our findings further indicate an important functional crosstalk between bitter taste receptor signaling and β-adrenergic pathways. Specifically, we demonstrate that HC inhibits the cAMP/PKA signaling cascade, thereby suppressing lipid droplet breakdown in Hep3B cells. This result is consistent with the clinical evidence showing that Cushing’s syndrome, characterized by excessive levels of HC and other steroid hormones, is associated with a markedly increased incidence of metabolic dysfunction-associated fatty liver disease (MAFLD) [43].

To further verify TAS2Rs’ role in regulating cAMP production, PKA substrate phosphorylation and lipolysis, we used siRNA-mediated knockdown to suppress TAS2R expression (Figure 3, Figure 5, Figure 6 and Figure 8). Our results indeed confirmed TAS2Rs as the receptors responsible for the bitter-compound-induced inhibition on cAMP production, PKA substrate phosphorylation and LD degradation. However, among the three siRNAs tested in Hep3B cells, only siTAS2R46-1 produced a significant effect in functional assays. In contrast, all three siRNAs used in MPHs exhibited consistent effects across different experiments. Notably, MPH cells are terminally differentiated and do not proliferate any more in vitro. As a result, intracellular siRNA concentrations are not diluted by cell division, which may account for the more stable and efficient knockdown observed in the functional assays compared with those in Hep3B cells.

Having established TAS2Rs’ contribution to the regulation of lipid metabolism in hepatic cells and primary hepatocytes, we further used a mouse model to validate the inhibitory effect of bitter compounds on LD breakdown in the liver (Figure 9). A fasting-induced hepatic steatosis mouse model has been widely used in animal intervention studies [29]. In recent years, fasting has been reported to exert beneficial regulatory effects on metabolic processes in both humans and animals [44]. Intermittent fasting regimens, such as the 16:8 diet, have also gained popularity as health-promoting lifestyle strategies [45]. And diets enriched with bitter compounds have been proposed to confer metabolic benefits [46]. Our results indicate that bitter compound treatments increase lipid droplets and delay TAG degradation, suggesting that although both fasting and bitter compound intake individually exert positive metabolic effects, their simultaneous application may lead to unexpected outcomes. These findings highlight the potential complexity of metabolic regulation when multiple interventions are combined.

In conclusion, this study demonstrates that TAS2Rs are expressed in the bile canaliculi of hepatocytes, where they co-localize with β-adrenergic receptors. Upon activation by bitter compounds, these receptors exhibit ligand-dependent coupling preferences to Gαi1, Gαi2, and Gαi3 subunits, leading to the direct inhibition of the βAR-mediated cAMP-PKA signaling pathway. Consequently, TAS2R activation significantly attenuates lipid droplet degradation and lipolysis in both hepatic cell models and an in vivo mouse model. These findings establish TAS2Rs as important modulators of hepatic lipid metabolism, providing a mechanistic basis for targeting this receptor-signaling axis in the management of metabolic dysfunction-associated fatty liver disease.

## 4. Materials and Methods

### 4.1. Materials

Denatonium benzoate (DB), isoproterenol (ISO), and hydrocortisone (HC) were purchased from Aladdin (Shanghai, China); oleic acid, quinine (Q), and saccharin (SAC) were obtained from Sangon Biotech (Shanghai, China); aloin (ALO), cromolyn (CRO), and diphenidol (DIP) were purchased from MedChemExpress (Shanghai, China); and aristolochic acid sodium salt (AA) was purchased from Sigma-Aldrich (Darmstadt, Germany). Human liver sections were purchased from Wenyuan Education Instrument and Equipment Corp (Xinxiang, Henan, China). Rabbit polyclonal antibodies against TA2R38 (PA5-99928, 1:100), TAS2R31 (PA5-99955, 1:100), and TAS2R46 (PA5-99956, 1:100) were purchased from Invitrogen (Carlsbad, CA, USA). Rabbit polyclonal antibody against TAS2R4 (NB110-74890, 1: 100) and mouse monoclonal antibodies against TAS2R3 (sc-398489, 1:50) and BSEP (67512-1, 1:500; 18990-1, 1:500) were purchased from Novus Biologicals (Centennial, CO, USA), Santa Cruz Biotechnology (Dallas, TX, USA), and Proteintech (Wuhan, Hubei, China), respectively, whereas rabbit polyclonal antibodies against β1AR (bs-0498R, 1:100) and β2AR (bs-0947R, 1:100) were purchased from Bioss (Beijing, China). Anti-rabbit IgG secondary antibodies (H&L, Alexa Fluor 488, ab150077, 1:500; Alexa Fluor 568, ab175471, 1:500) and anti-mouse IgG secondary antibodies (H&L, Alexa Fluor 488, ab150113, 1:500; Alexa Fluor 568, ab175472, 1:500) were purchased form Abcam (Cambridge, UK).

### 4.2. Plasmid Construction

Expression plasmids for bitter taste receptors TAS2R14 and TAS2R31 were derived from previous studies [18]. The coding sequences for bitter taste receptors TAS2R20 and TAS2R46 were amplified from cDNA of Hep3B cells. To promote expression and membrane localization of bitter taste receptors, a 45-amino acid signal peptide sequence from the N-terminus of rat somatostatin 3 (SSTR3) was inserted upstream of the coding region. The TRUPATH Triple Gai1, Gai2 and Gai3 plasmids were purchased from Addgene (Watertown, MA, USA). All plasmids used in this study were verified by DNA sequencing.

### 4.3. Cell Cultures and Animals

Hep3B2.1-7 and HEK293T were purchased from Procell Life Science & Technology Co., Ltd. (Wuhan, China). Hep3B2.1-7 was maintained in minimum essential medium (MEM) supplemented with 10% FBS (Gibco, Grand Island, NY, USA, 10270106), 2 mM GlutaMAX, 1× NEAA and 100 U/mL penicillin–streptomycin. HEK293T was maintained in Dulbecco’s Modified Eagle’s Medium (DMEM) supplemented with 10% FBS (Gibco, 10270106), 2 mM GlutaMAX, and 100 U/mL penicillin–streptomycin. C57BL/6 mice were purchased from Hangzhou Medical College (Hangzhou, China). Studies involving animals were approved by the Zhejiang University Institutional Animal Care and Use Committee and performed following the NIH Guidelines for the Care and Use of Laboratory Animals.

### 4.4. Isolation and Collagen Sandwich Culture of Mouse Hepatocytes

The isolation of mouse primary hepatocytes (MPHs) was performed according to the previously described protocol [47]. Briefly, the mouse was anesthetized, and its liver was retro-perfused from the inferior vena cava to the portal vein with the EDTA-containing perfusion buffer followed by the digestion buffer containing the liberase (Roche, Penzberg, Germany, 5401119001). Then, the liver was transferred to a plate and ruptured with fine-tipped forceps to release dissociated cells, which were washed by centrifugation, and then purified by Percoll-based density separation (Solarbio, Beijing, China, P8370). The viability of the cells was checked using trypan blue staining.

To induce hepatocytes to form bile canaliculi, collagen sandwich culture was performed according to the previously published protocols [48,49]. Briefly, the isolated hepatocytes were plated in a dish pre-coated with 1.5 mg/mL collagen type I (Corning, Corning, NY, USA, 354249). The hepatocytes were plated onto the collagen gel and overlaid with another layer of collagen type I. Cells were cultured in the hepatocyte culture medium (DMEM high glucose plus 10% FBS, 2 mM GlutaMAX, 1× penicillin–streptomycin, 0.3 μg/mL glucagon, 15 μg/mL hydrocortisone and 8 μg/mL insulin). The medium was refreshed daily until the bile canaliculi were formed among adjacent hepatocytes.

### 4.5. RNA Extraction and Quantitative RT-PCR

Total RNA was extracted from Hep3B2.1-7 cells, MPHs, and adipose tissue using the Trizol reagent (Invitrogen, 15596026), which was further purified using another column-based RNA Extraction Kit (Takara, Shiga, Japan, 9767) to eliminate any residual genomic DNA. Approximately 3 μg of total RNA was used for reverse transcription using the PrimeScript II 1st Strand cDNA Synthesis Kit (Takara, 6210). Quantitative PCR reactions were set up using the primers listed in Supplementary Table S1 and iTaq Universal SYBR Green Supermix (Bio-rad, Hercules, CA, USA, 1725124) and performed on the CFX Connect Real-Time System. Human *HPRT1* or mouse *Hprt1* was used as an internal “housekeeping” control gene for normalization between different samples, and relative expression levels of target genes were calculated using the 2^−ΔΔCt^ method [50,51].

### 4.6. Immunofluorescence (IF) Staining

IF staining was performed as previously described with slight modification [52]. The liver was fixed with 4% paraformaldehyde (PFA) overnight and then cryoprotected in 30% (*w*/*v*) sucrose for 24 h. After embedded in the O.C.T. medium (Tissue-Tek, Torrance, CA, USA), the tissue was sliced into 14 μm thick sections on a cryostat (Thermo scientific, Waltham, MA, USA, HM525). The sections were then washed with 1× PBS and blocked in the blocking buffer (3% BSA, 0.3% Triton X-100, 2% horse serum and 0.1% sodium azide in 1× PBS) for 1 h. To immunofluorescently stain cultured cells, the cells were fixed with 4% PFA at room temperature for 30 min and then washed with 1× PBS and blocked in the blocking buffer for 1 h. Both the tissue sections and cultured cells were incubated with primary antibodies at 4 °C overnight, followed by the corresponding secondary antibodies at room temperature for 1 h. To immunostain the actin web around the bile canaliculi, Alexa Fluor 594-conjugated phalloidin (Beyotime, Shanghai, China, C2205S) was added along with the secondary antibodies. DAPI was used for nuclear staining (Sigma-Aldrich, D9542). Fluorescent images were captured using an Olympus FV3000 confocal laser-scanning microscope.

### 4.7. Live Cell Imaging with CFDA

To determine the transport function of the bile canaliculi, we perform the CFDA assay as described previously [53]. Briefly, the cells for imaging were seeded onto a 35 mm glass-bottom cell culture dish. Three days later, the culture medium was washed with Hank’s Balanced Salt Solution (HBSS) to remove any residual serum. Cells were then cultured in the HBSS containing 5 μM 5(6)-carboxy-fluorescein diacetate (CFDA) (Sigma-Aldrich, 21879) and 5 μg/mL Hoechst 33342 (Sigma-Aldrich, B2261). Fluorescent images of the cells were taken using an Olympus IX83 fluorescence inverted microscope equipped with a set of filters for 494/521 nm of excitation–emission wavelengths every 10 s for 30 min to examine the accumulation of CFDA in the bile canalicular lumens.

### 4.8. Cell Viability Assay

Cells were seeded in a 96-well plate at a density of 20,000 cells per well. Twenty-four hours after seeding, the culture medium was replaced with the medium containing different concentrations of bitter substances, with each concentration tested in six replicates. After 24 h culture in the medium containing the bitter substances, the medium was replaced with the one without the bitter substances to avoid any possible interferences from these substances on the cell viability assay. Cells were then incubated in a culture medium containing a 10% CCK-8 solution (Biosharp, Beijing, China, BS350) for 30 min, and absorbance was measured at 450 nm. The cell viability was determined following the manufacturer’s instructions.

### 4.9. Bioluminescence Resonance Energy Transfer (BRET) Assay

Bioluminescent resonance energy transfer (BRET) experiments were performed in HEK293T cells according to a published protocol [54]. About 2.5 million cells were seeded into a 60 mm culture dish with a total culture medium volume of 4 mL. After overnight culture, cells were co-transfected with 2 μg of TRUPATH Triple Gai1, Gai2, or Gai3 plasmids and 2 μg of TAS2R14, TAS2R20, TAS2R31, or TAS2R46 expression plasmids using NB Trans transfection reagent. After 24 h, cells were digested into single cells using the Versene solution, counted, and seeded into 40,000 cells per well in white-bottomed 96-well plates coated with poly-L-lysine.

Twenty-four hours after cells being seeded into the 96-well plates, all culture medium was discarded, and 70 μL of detection buffer (HBSS solution containing 20 mM HEPES) was added to each well. Before chemiluminescence detection, 20 μL of Coelenterazine 400 solution (25 μM, dissolved in the detection buffer) was added, resulting in a final concentration of 5 μM. Chemiluminescence detection was performed in a Varioskan LUX microscope with filters at 425 nm and 515 nm wavelengths. The integration time was 1 s, and detection began 2 min after the addition of the substrate. Chemiluminescence signals were detected at two wavelengths sequentially. Subsequently, 10 μL of bitter stimuli dissolved in the detection buffer was added, and after equilibration for 2 min, the second scans were performed.

The BRET ratio is the emission value at 515 nm divided by that at 425 nm. To eliminate the influence of cell mass and the bitter substance itself on the BRET ratio of each well, the BRET ratio of the bitter substance sample well needs to be subtracted successively from the baseline value before the addition of the bitter substance and the BRET ratio of the single donor transfection sample with the same concentration of the bitter substance. The resulting value is then subtracted from the BRET difference of the solvent well without the bitter substance and normalized to obtain the ∆BRET value of the corresponding well.

### 4.10. cAMP Assay

cAMP assay was performed according to the previously reported protocols with some modifications [55,56]. Briefly, MPHs used for cAMP assay were grown in a 35 mm cell culture dish, and on the day before experiment, insulin, glucagon, and hydrocortisone in the hepatocyte culture medium were removed. On the following day, IBMX (Sigma-Aldrich, 410957) and Ro-20-1724 (Sigma-Aldrich, 557502) were added to the medium right before the experiment to inhibit the activity of cyclic nucleotide phosphodiesterases. Thirty minutes after stimulation, the medium was removed, and the culture dishes were directly frozen at −80 °C until use. The amount of intracellular cAMP was determined using a cAMP Select ELISA Kit (Cayman, Ann Arbor, MI, USA, 501040) according to the manufacturer’s instructions. The same protocol was used to determine the intracellular cAMP concentrations in the Hep3B2.1-7 cells after the cells were grown in 60 mm cell culture dishes to 70% confluency.

### 4.11. Western Blot Analysis

The Western blot procedures were performed as previously described [57]. Briefly, the cells were collected after being treated with corresponding stimuli for 1 h and lysed with the RIPA lysis buffer containing Halt Protease and Phosphatase Inhibitor Cocktail (Thermo Scientific, 78440) on ice for 30 min. After centrifugation, the supernatants were collected and boiled for 5 min for denaturation. The samples were then loaded into 10% SAD-PAGE gel and transferred to PVDF membranes (Millipore, Burlington, MA, USA, IPVH00010). After blocking with 5% BSA, the membrane was incubated at 4 °C overnight with primary antibodies diluted in the blocking buffer (anti-phospho-PKA substrate antibody, Cell Signaling, Danvers, MA, USA, 9624; anti-phosphor-HSL antibody, Cell Signaling 4139; anti-HSL antibody, Cell Signaling 4107; anti-GAPDH antibody, Proteintech, Wuhan, Hubei, China, 60004-1-Ig). For chemiluminescent detection, the membrane was incubated with corresponding secondary antibodies conjugated to HRP. The images were taken with the Tanon 5200 Chemiluminescent Imaging System and quantified using ImageJ.

### 4.12. siRNA Transfection

The procedure for siRNA transfection of MPHs was optimized based on a previously reported protocol [58]. Briefly, 30 min before the transfection, the cell culture medium was replaced by the opti-MEM Reduced Serum Medium (Gibco, 31985062). And 100 nmol siRNA (Tsingke, Beijing, China) and 8 μL Lipofectamine RNAiMAX (Invitrogen, 13778100) reagent were used to transfect cells in a 35 mm culture dish. Six hours post-transfection, the opti-MEM medium was removed, and a layer of collagen was applied on the top of the cells. HCM medium was added after collagen solidified as described above for the collagen sandwich culture. The siRNA sequences are provided in Supplementary Table S2.

### 4.13. Fasting and Refeeding Mouse Model

The mouse model was established according to a previously described method [29]. Briefly, bodyweight-matched littermates were individually housed for 24 h to acclimate to the new environment, followed by another 24 h of food deprivation. The specific dosages for bitter stimuli were determined based on the safety data of LD50 for each chemical from the PubChem database [59]. Initial preliminary in vivo tests were also performed to identify the minimal effective doses that robustly blunt hepatic lipolysis without causing overt toxicity or behavioral abnormalities in the mice. Based on these assessments, the mice were intraperitoneally injected with DB (5 mg/kg), DIP (15 mg/kg), Q (15 mg/kg), SAC (400 mg/kg) or an equal volume of 1× PBS as a control and then refed with regular chow for 6 h. The livers were harvested after cardiac perfusion with 1× PBS to remove excess blood. The liver tissues were flash frozen and stored until being used for oil red O staining or quantification of cellular TAG.

### 4.14. Bodipy Staining and Oil Red O Staining Analyses

Bodipy staining and oil red O (ORO) staining were used to analyze lipid droplets (LDs) in the cultured hepatocytes and the liver tissue sections, respectively. Bodipy staining was performed following a previously described protocol [60]. For MPHs, the bile canaliculi were generated using the aforementioned collagen sandwich culture method. One day before the treatment, the cells were incubated with 200 μM oleic acid (OA). Then, the cells were treated with the indicated stimuli in the absence of OA for 24 h. Afterwards, the cells were washed with 1× PBS and fixed with 4% PFA for 30 min at room temperature and then stained with the fluorescent neutral lipid dye 4,4-difluoro-1,3,5,7,8-pentamethyl-4-bora-3a,4a-diaza-s-indacene (Bodipy 493/503) (Invitrogen, D3922). DAPI was used for nuclear staining. Fluorescent images were captured using an Olympus FV3000 confocal laser-scanning microscope (Tokyo, Japan). The LD analysis was performed using the software ImageJ (version 1.54) [61].

The ORO staining was performed as previously described to assess cellular lipid contents in the liver tissue sections [62]. Briefly, mouse liver tissue was sliced into 12 μm thick sections, which were incubated with the filtered ORO working solution. The sections were rinsed with running tap water for 30 min to remove any nonspecific background staining. The sections were mounted in glycerol–PBS and sealed for imaging. Bright-field images were captured with a 3Dhistech Pannoramic MIDI II Digital Slide Scanner (Budapest, Hungary) and analyzed with the software ImageJ.

### 4.15. Quantification of Glycerol and FFA in the Culture Medium

The quantification of glycerol and FFA was modified from the previously published methods for cultured adipocytes [63]. The cells were first pretreated with 200 μM OA for 24 h to induce LD formation and then serum-starved for 2 h to eliminate any possible effect from insulin or growth factors present in the serum. The medium then was replaced with serum-free medium containing 2% FFA-free BSA and desired stimuli and incubated for 2 h. The culture medium was collected and used for the measurement of glycerol and FFA with an Amplex Red Glycerol Assay Kit and Amplex Red Free Fatty Acid Assay Kit (Beyotime, S0223 and S0215). The cells were also collected and lysed with the RIPA lysis buffer and used for the quantification of protein amount with a BCA protein assay kit (Sangon, Shanghai, China, C503021). The production of glycerol and FFA was expressed as nmol glycerol/FFA per mg protein per hour and used for statistical analysis.

### 4.16. Quantification of Triglyceride (TAG) Contents in Mouse Liver

The quantitation of cellular triglycerides was performed as previously described [64]. Briefly, a piece of liver tissue was retrieved from −80 °C storage and digested overnight at 55 °C with a KOH–ethanol solution until the tissue was fully digested and no oil layer was visible. The homogenate was centrifuged briefly to remove any insoluble contents. The supernatant was transferred and treated with MgCl_2_ to salt out free fatty acids. Following another centrifugation, the supernatant was collected and subjected to triglyceride measurement, which was performed according to the manufacturer’s instruction (Sigma-Aldrich, F6428).

### 4.17. Statistical Analysis

Data are expressed as mean ± SD from at least 3 independent experiments. The homogeneity of SDs and normality of data distribution were verified with the Brown–Forsythe test and Shapiro–Wilk test, respectively. Significance was determined with one-way ANOVA followed by Dunnett’s multiple comparisons test or two-way ANOVA followed by Tukey’s multiple comparisons test. Statistical analyses were all performed in GraphPad Prism 9.

## Figures and Tables

**Table 1:** Reported concentration (μM) of each bitter stimulus used to effectively activate human bitter taste receptor(s).

	ALO	AA	CRO	DB	DIP	HC	Q	SAC
TAS2R31	30	0.016			3		10	80
TAS2R43	0.3	1100	3000	300	30		10	170
TAS2R20			10		100			
TAS2R46				30	30	3	10	
TAS2R14		10.3			10		10	
TAS2R30				0.03	100			
TAS2R10				3	30		10	
TAS2R4				300	100		10	
TAS2R13				30	30			
TAS2R7			3000		10		10	
TAS2R39				100	100		10	
TAS2R8				1000				

Data in the table were obtained from the database BitterDB [20]. AA, aristolochic acid; ALO, aloin; CRO, cromolyn; DB, denatonium benzoate; DIP, diphenidol; HC, hydrocortisone; Q, quinine; SAC, saccharin.

**Table 2:** Reported concentration (μM) of each bitter stimulus used to effectively activate mouse bitter taste receptor(s).

	ALO	AA	CRO	DB	DIP	HC	Q	SAC
Tas2r126							10	
Tas2r137					100		10	
Tas2r135				100				100
Tas2r108					100		10	
Tas2r140				300			3	

Data in the table were obtained from the database BitterDB [20]. AA, aristolochic acid; ALO, aloin; CRO, cromolyn; DB, denatonium benzoate; DIP, diphenidol; HC, hydrocortisone; Q, quinine; SAC, saccharin.

## Data Availability

The original contributions presented in this study are included in the article/Supplementary Material. Further inquiries can be directed to the corresponding author(s).

## Supplementary Materials

The following supporting information can be downloaded at https://www.mdpi.com/article/10.3390/ijms27073226/s1.

## Abbreviations

The following abbreviations are used in this manuscript:

AA	Aristolochic acid
AC	Adenylyl cyclase
ALO	Aloin
ATGL	Adipose triglyceride lipase
BSEP	Bile salt export pump
BRET	Bioluminescence resonance energy transfer
cAMP	Cyclic adenosine monophosphate
CFDA	5(6)-carboxy-fluorescein diacetate
CRO	Cromolyn
DB	Denatonium benzoate
DIP	Diphenidol
DMSO	Dimethyl sulfoxide
FFA	Free fatty acid
GPCR	G protein-coupled receptor
HC	Hydrocortisone
HSL	Hormone sensitive lipase
IF	Immunofluorescence
ISO	Isoproterenol
LD	Lipid droplet
MAFLD	Metabolic dysfunction-associated fatty liver disease
MPH	Mouse primary hepatocytes
ORO	Oil red O
PKA	Protein kinase A
PLIN1	Perilipin-1
Q	Quinine
SAC	Saccharin
siRNA	Small interfering RNA
TAG	Triacylglycerol
TAS2R	Type 2 taste receptor.
αAR	α-Adrenergic receptor
βAR	β-Adrenergic receptor
β1AR	β1-Adrenergic receptor
β2AR	β2-Adrenergic receptor
