# Supplementary material for: Bile Canalicular Bitter Taste Receptors Inhibit β-Adrenergic Receptor-Induced Lipolysis in Steatotic Hepatocytes
Source: Int J Mol Sci. 2026 Apr 2;27(7):3226. doi: 10.3390/ijms27073226 (PMC13073796; doi:10.3390/ijms27073226)
Supplement: Supplementary file 1 [file ijms-27-03226-s001.zip › ijms-4206344-supplementary.pdf]

Title

Bile canalicular bitter taste receptors inhibit β-adrenergic receptor-induced lipolysis in steatotic hepatocytes

Journal

International Journal of Molecular Sciences

Authors

Yan-Bo Xue [1], Shi-Meng Gong [1], Yuan-Yuan Peng [1], Defu Yu [1], Ruhong Zhou [1,2*], Liquan Huang [1,2*]

Affiliation

1: College of Life Sciences, Zhejiang University, Hangzhou, Zhejiang Province, China
2. Zhejiang University Shanghai Institute for Advanced Study, Shanghai, Shanghai, China

# Corresponding authors

Ruhong Zhou, rhzhou@zju.edu.cn
Liquan Huang, huangliquan@zju.edu.cn

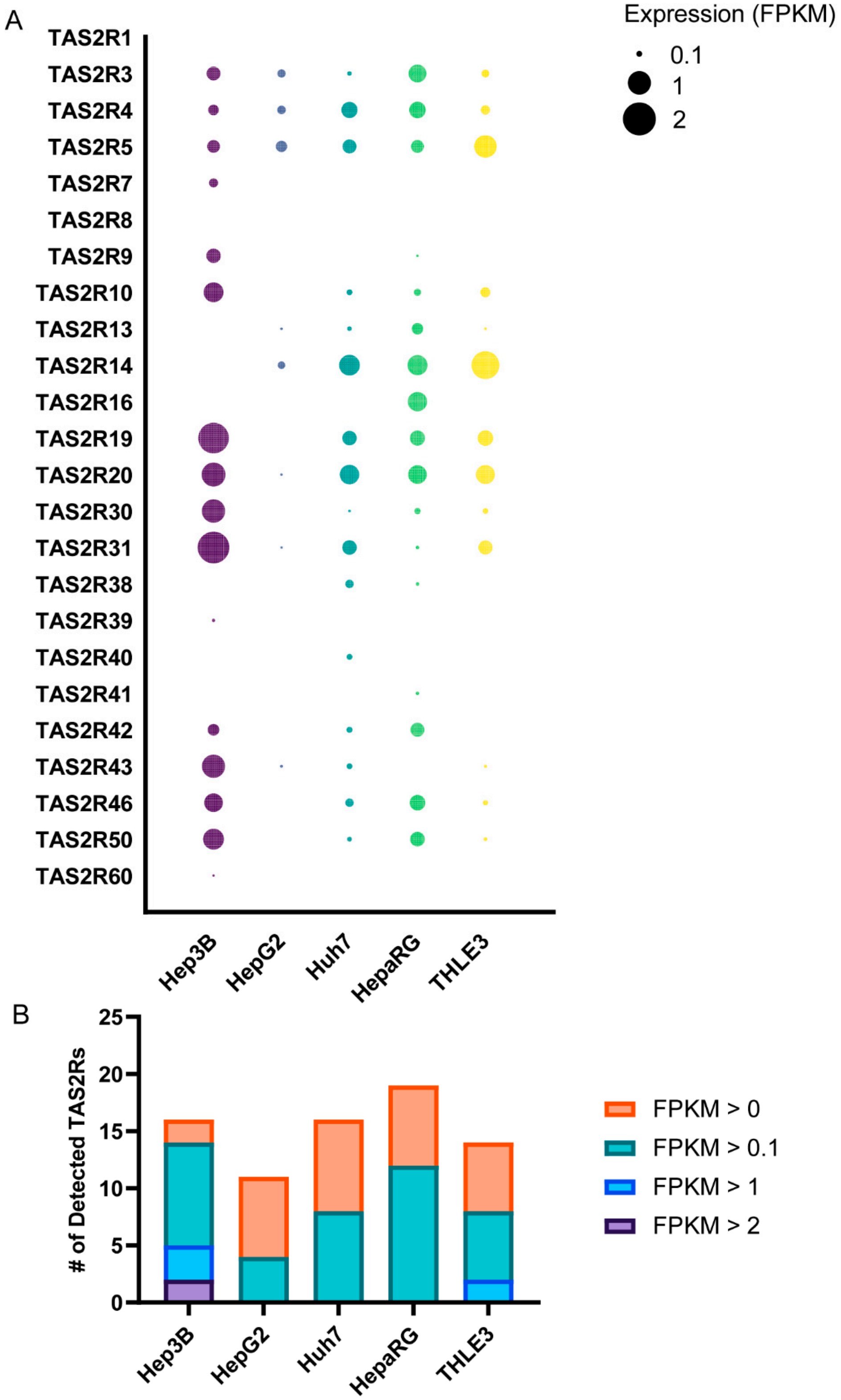

Figure S1. TAS2Rs expression in 5 human hepatic cell lines. (**A**) Gene expression analysis of all TAS2Rs in the RNAseq datasets of Hep3B, HepG2, Huh7, HpeaRG and THLE3 cells. (**B**) TAS2R genes detected in these RNAseq datasets were ranked according to their fragments per kilobase of transcript per million mapped reads (FPKM).

A

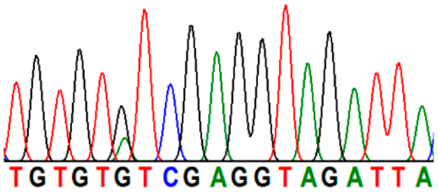

B

Hep3B TAS2R2: TGTGTGT- -CGAGGTAGATTA

Intact TAS2R2: TGTGTGTATCGAGGTAGATTA

Figure S2. *TAS2R2* sequencing and alignment result. (**A**) PCR product was amplified from Hep3B cDNA using the primers TAS2R2F and TAS2R2R. Sequencing was performed using the primer TAS2R2R, and the region containing the reported deletion is shown. (**B**) Comparison between the *TAS2R2* gene sequence in Hep3B cells and the intact *TAS2R2* gene sequence revealed a deletion. The two deleted bases are highlighted in the intact reference sequence.

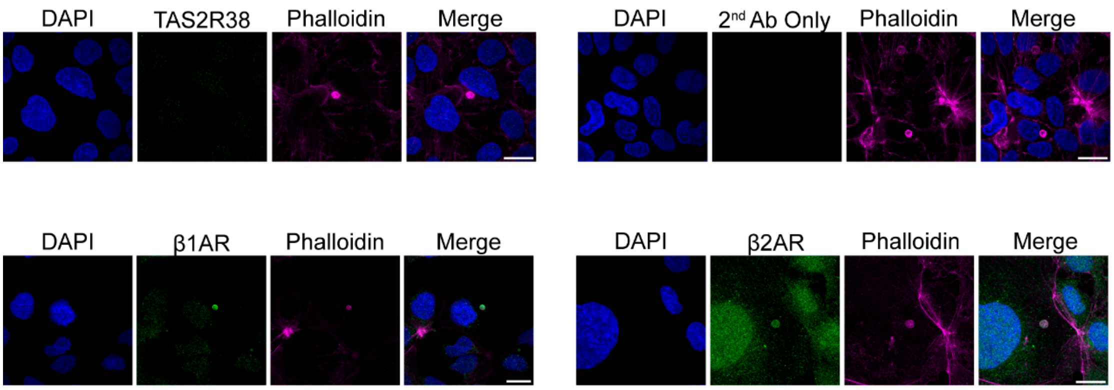

Figure S3. Immunofluorescent images of TAS2R38, β1AR, and β2AR in Hep3B cells. Negative control (TAS2R38, top left) and technical control (2nd Ab only, top right) showed no detectable green fluorescent signal whereas the green fluorescent signals for β1AR and β2AR (bottom left and right, respectively) were localized to the pseudo-canaliculi (magenta). DAPI (blue) was used for nuclear counterstaining. Phalloidin was used to label the actin-rich pseudo-canaliculi (magenta) formed by Hep3B cells. Scale bar: 20 μm.

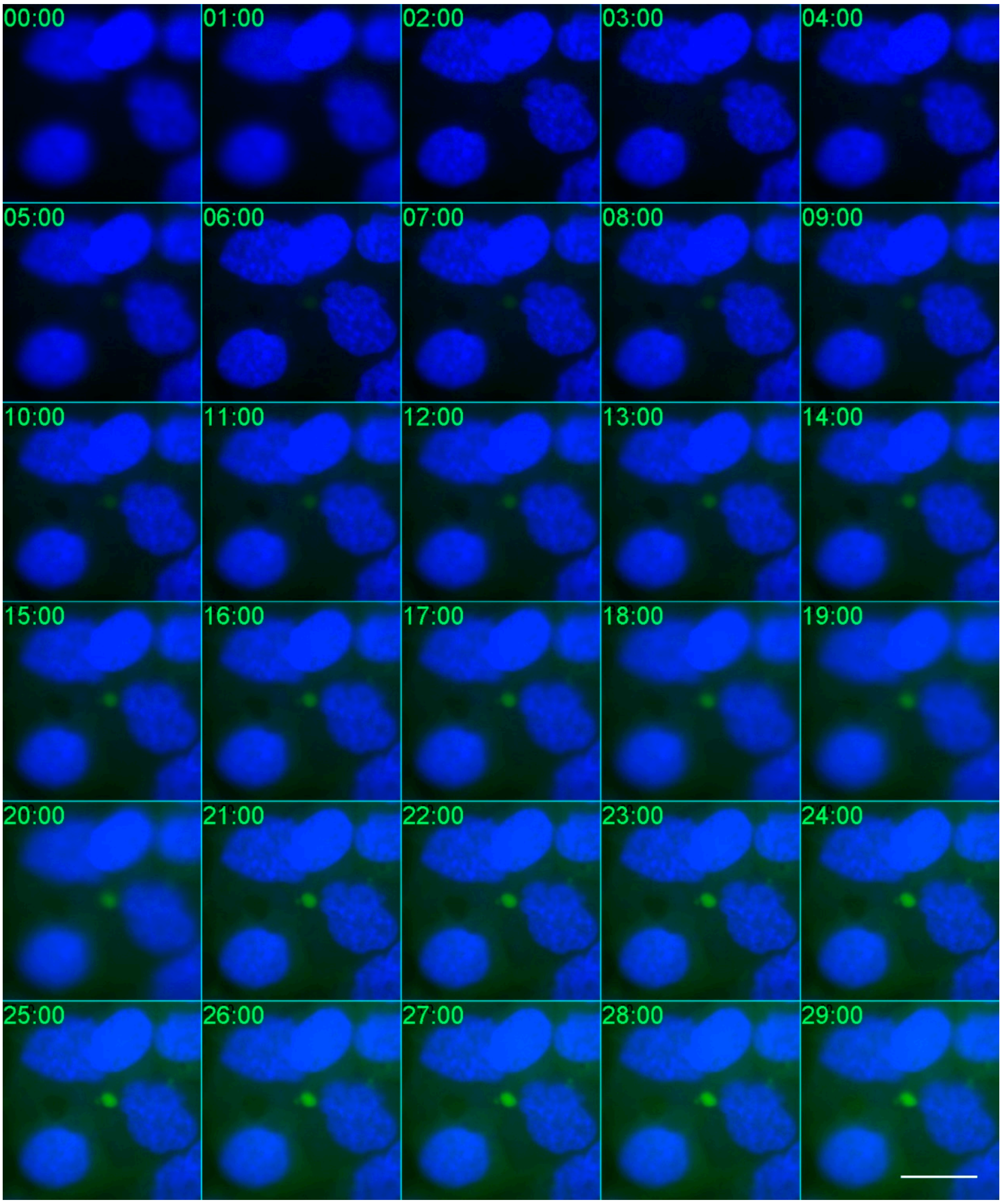

Figure S4. Evaluation of carboxy-fluorescein diacetate (CFDA) transport and accumulation in the lumen of pseudo-bile canaliculi. In the pseudo-canaliculus formed by Hep3B cells, CFDA was directionally transported into its enclosed central lumen, which is shown here as a green dot in the center of the images. The time point is indicated in the upper left corner of each image (mm:ss). Scale bar: 20 µm

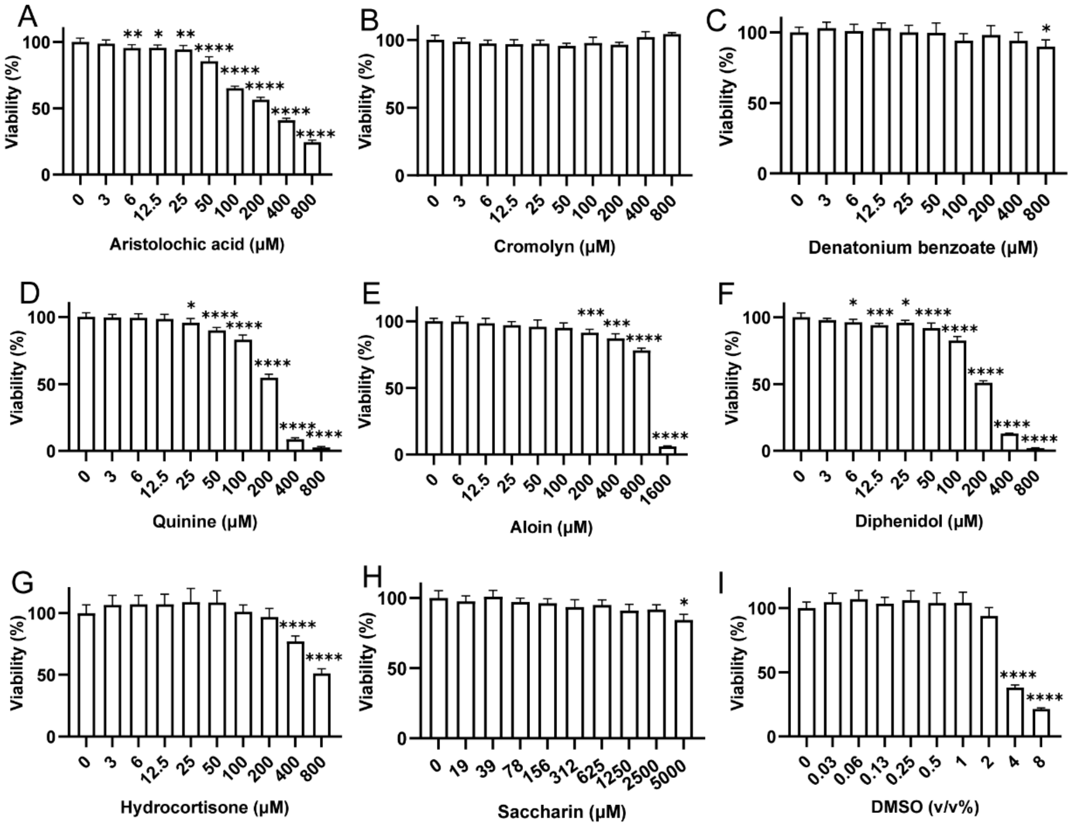

Figure S5. Determination of optimal bitter compound working concentrations with minimal cytotoxicity on Hep3B cells. (**A-D**) Water-soluble bitter stimuli aristolochic acid (AA) (**A**), cromolyn (CRO) (**B**), denatonium benzoate (DB) (**C**) and quinine (Q) (**D**) were applied to Hep3B for 24 hours at the indicated concentrations. (**E-H**) DMSO-soluble bitter stimuli aloin (ALO) (**E**), diphenidol (DIP) (**F**), hydrocortisone (HC) (**G**) and saccharin (SAC) (**H**) were also applied to Hep3B for 24 hours at the indicated concentrations. (**I**) DMSO was also used to treat Hep3B to rule out the toxicity that drug solvent may bring. Cell viability was measured using the cell counting kit-8. n=6; ∗: $p < 0.05$; ∗∗: $p < 0.01$; ∗∗∗: $p < 0.001$; ∗∗∗∗: $p < 0.0001$.

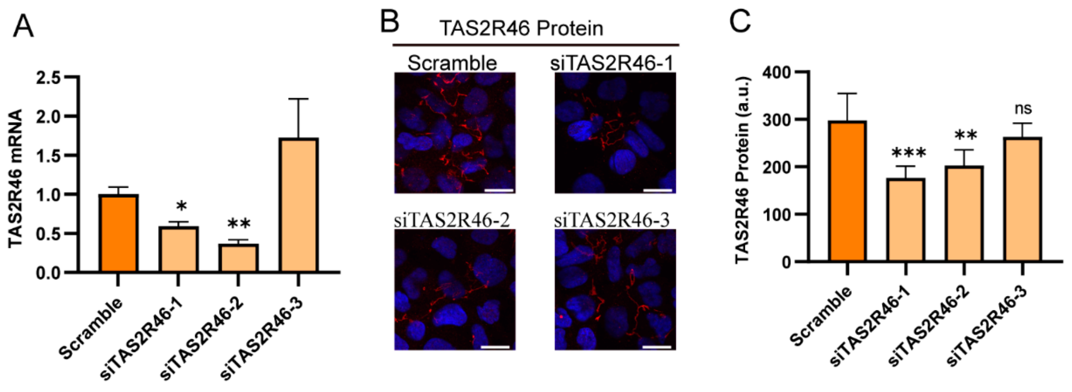

Figure S6. Validation of TAS2R46 siRNA knockdown efficiency in Hep3B cells. (**A**) Knockdown efficiency of *TAS2R46* was assessed by qRT-PCR (n=3). (**B**) TAS2R46 protein levels were assessed by immunofluorescent (IF) staining 48 hours post siRNA transfection. (**C**) Quantification of TAS2R46 IF signals from (**B**) (n=5). ns: no statistically significance; ∗: $p < 0.05$; ∗∗: $p < 0.01$; ∗∗∗: $p < 0.001$; ∗∗∗∗: $p < 0.0001$; Scale bar: 20 μm.

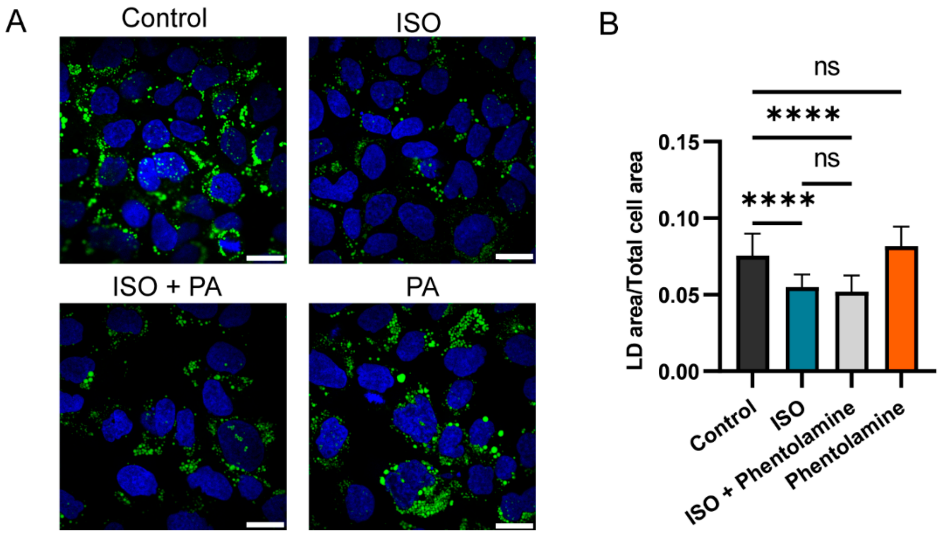

Figure S7. Effect of the αAR antagonist phentolamine on βAR-induced lipolysis in Hep3B cells. (**A**) Representative images of Hep3B cells treated for 24 h with 50 µM isoproterenol (ISO), 50 µM ISO + 25 µM phentolamine (PA), or 25 µM PA alone. Control cells were treated with DMSO. (**B**) Lipid droplet (LD) areas in panel **A** were quantified and normalized to cell area. n>10; ns: no statistical significance; ****: $p < 0.0001$. Scale bar: 20 µm.

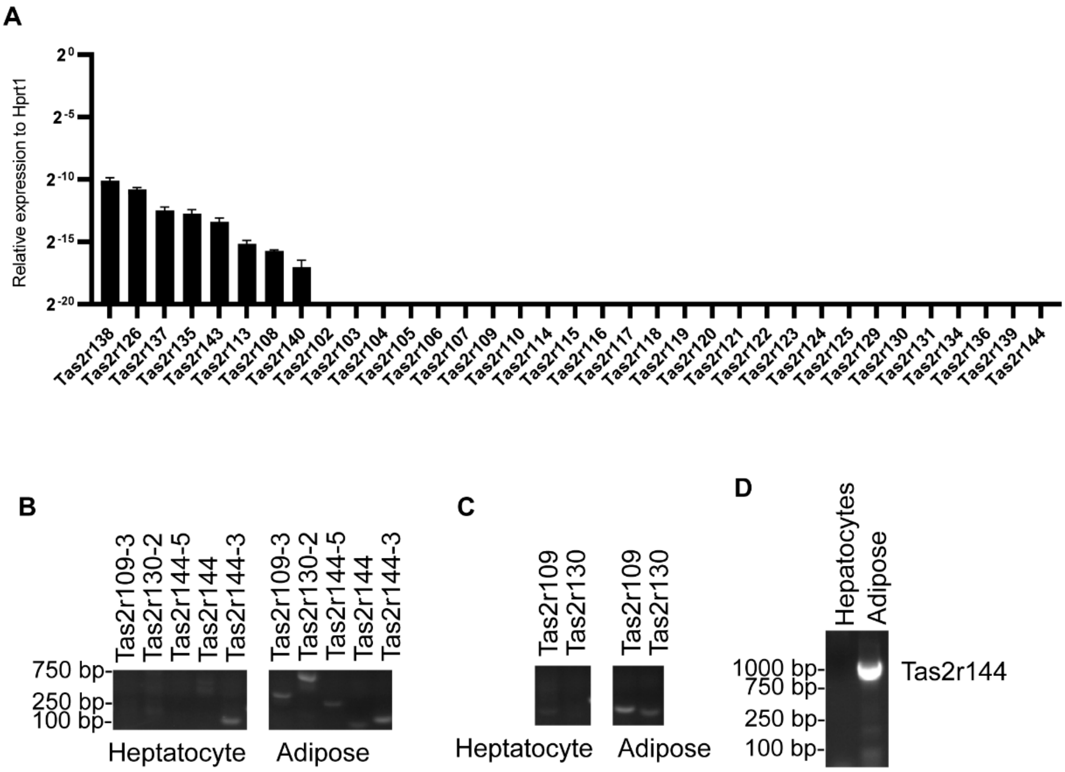

Figure S8. Gene expression profiling of all 35 Tas2r genes in mouse primary hepatocytes. (**A**) Expression levels were calculated as relative ratios of Tas2r genes versus the internal control gene *Hprt1*. (**B-C**) PCR primers of *Tas2r109*, *Tas2r130*, *Tas2r144* were validated using cDNAs derived from hepatocytes and adipose tissue. Primer sequences for the PCR reactions shown in panel **B** were from previously reported literature, while those in panel **C** were from another study. (**D**) Full-length transcripts of *Tas2r144* were successfully amplified from adipose cDNA, but not from hepatocyte cDNA.

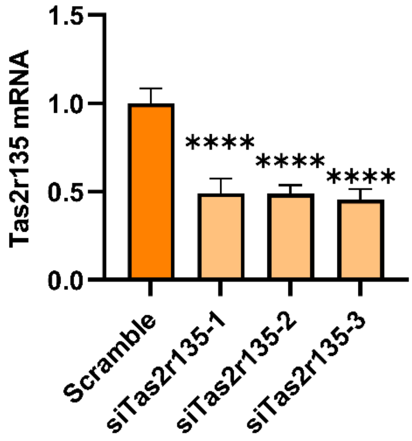

Figure S9. Validation of *Tas2r135* siRNA knockdown efficiency in mouse primary hepatocytes. The knockdown efficacy was assessed using qRT-PCR. n=3; ****: $p < 0.0001$.

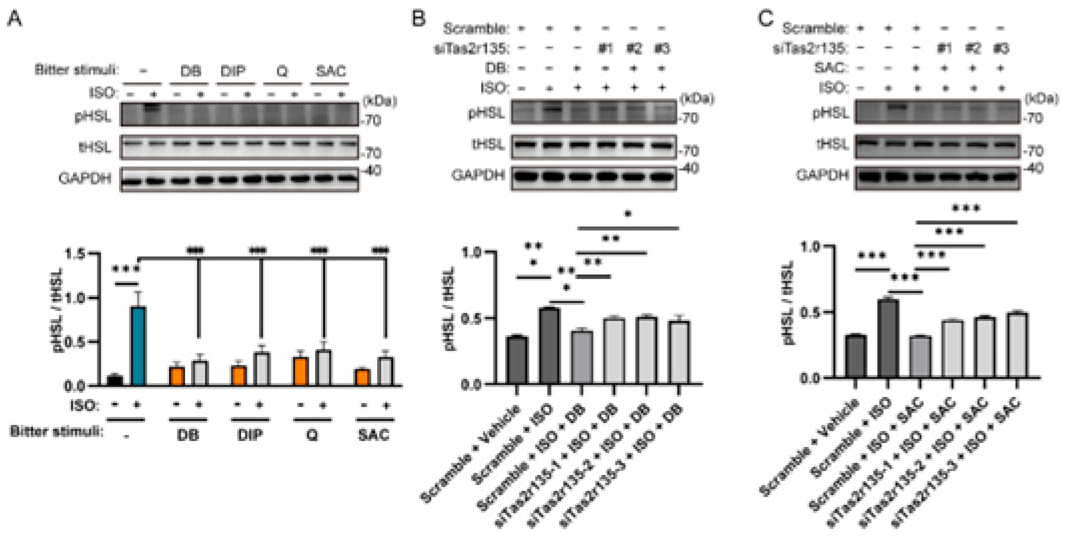

Figure S10. Effect of bitter compounds on isoproterenol (ISO)-induced hormone sensitive lipase (HSL) phosphorylation in mouse primary hepatocytes (MPH). (**A**) Western blot analysis of phosphorylated HSL (pHSL) versus total HSL (tHSL) in Hep3B cells treated with ISO (50 μM) and one of the four bitter stimuli (100 μM DB, 100 μM DIP, 25 μM Q, or 2 mM SAC) alone or in combination. (**B**) Western blot analysis of phosphorylated HSL (pHSL) versus total HSL (tHSL) in *Tas2r135* siRNA-transfected MPH treated with ISO (50 μM) and DB (100 μM) alone or in combination. (**C**) Western blot analysis of phosphorylated HSL (pHSL) versus total HSL (tHSL) in *Tas2r135* siRNA-transfected MPH treated with ISO (50 μM) and SAC (2 mM) alone or in combination. DB, denatonium benzoate; DIP, diphenidol; Q, quinine; SAC, saccharin. ∗: $p < 0.05$; ∗∗: $p < 0.01$; ∗∗∗: $p < 0.001$.

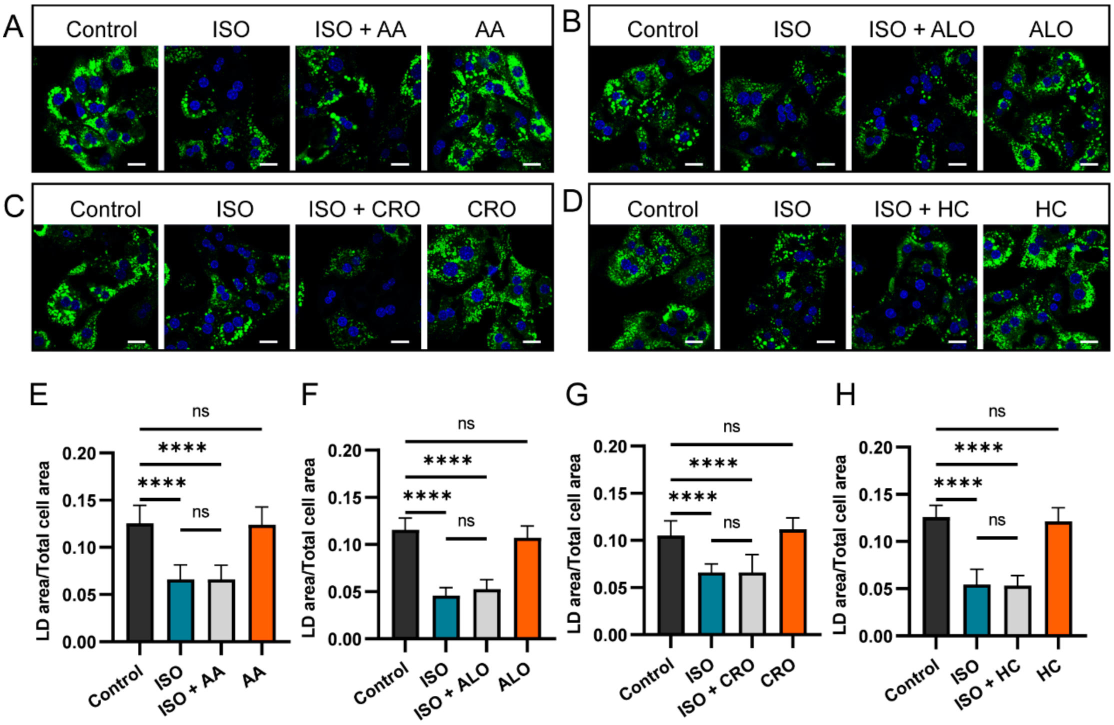

Figure S11. Evaluation of the effect of bitter compounds with no corresponding Tas2rs on βAR-mediated lipolysis in mouse primary hepatocytes (MPH). (**A-D**) Representative images showing green lipid droplet (LD) signal in MPH after 24-hour treatment with: 50 µM isoproterenol (ISO), 50 µM ISO + 25 µM AA, or 25 µM AA alone (**A**); 50 µM ISO, 50 µM ISO + 100 µM ALO, or 100 µM ALO alone (**B**); 50 µM ISO, 50 µM ISO + 100 µM CRO, or 100 µM CRO alone (**C**); 50 µM ISO, 50 µM ISO + 100 µM HC, or 100 µM HC alone (**D**). Control cells were treated with the solvent for each bitter compound. (**E-H**) Quantitative analysis of LD areas from images in panel **A-D**, normalized against the cell areas. n>10; ns: no statistical significance; ****: $p < 0.0001$. AA, aristolochic acid; ALO, aloin; CRO, cromolyn; HC, hydrocortisone. Scale bar: 20 µm.

Table S1 Sequences of PCR primers used in this study

Primer name	Sequence	Product length
HPRT1F	CTGGAAAGAATGTCTTGATTGTG	104
HPRT1R	GACCTIGACCATCTTIGGATTA	
TAS2R1F	ATCATGTGTTCTGCGAATTGTGC	230
TAS2R1R	ACCATAAACCCTGCATATTTGCT	
TAS2R3F	TGGCACTCTTGAGGATCATTCT	187
TAS2R3R	CTGGCGATTTTCAGGCAGTAG	
TAS2R4F	TTCCTGAACTTGTGACTACGAGA	146
TAS2R4R	CCTCAAGGAGTGTATTAGCAAGG	
TAS2R5F	GGGCCTATAACCTGAGTCTCTG	196
TAS2R5R	ACAAGAAACACCACTAAAGGCA	
TAS2R7F	GCCACTGAGAATTTGAACGCT	201
TAS2R7R	TCGCCTGATATGTCTCCGCA	
TAS2R8F	AACTCTATGCTACCGGCAGTA	196
TAS2R8R	TGCTGCAATCTCTCCAAACTC	
TAS2R9F	TGCATTGACTGGCTCAAAAGA	155
TAS2R9R	CTTACTAGCACGCTATTGCCAT	
TAS2R10F	GGCAGATGCAATCGAATGTGA	159
TAS2R10R	GCAGTTTGTTTTCTCGCACAGT	
TAS2R13F	GAAAGTGCCCTGCCGAGTATC	140
TAS2R13R	AGGAGTTTATCGACTGAGGACA	
TAS2R14F	ATGGGTGGTGTCATAAAGAGCA	173
TAS2R14R	AGGCTAATTCGAGAGATTGCCA	
TAS2R16F	ACAGTGGGCATCAATGCTGAA	126
TAS2R16R	ACGGTAAGCAAGCTGTTTAACC	
TAS2R20F	GCTTGCTACTAGCCTCAGCA	158
TAS2R20R	ACGTGTGTTTCATCACAAGGTG	
TAS2R30F	TCCTTCAACCCACCCATTCA	144
TAS2R30R	AAGACACACAATGCCCCTCTT	
TAS2R31F	GACTGTAACCACGCTAGGAAACTT	189
TAS2R31R	ACGGCACATAACAAGAGGAAAAA	
TAS2R38F	CGTTTCTCTCACACCTTCCTG	187
TAS2R38R	GCCAGTTGAGCCTTGTATTGTTA	
TAS2R39F	ATTACTGGATTGATACCCTGGCT	167
TAS2R39R	AGACCGACCACATTGATCTCA	
TAS2R40F	TCTTGGCGCAGAAACCTGAA	143
TAS2R40R	TCCCAAGGATGCCAGTGATG	
TAS2R41F	CTTCTTCGTGTTGCTCTTTAGCC	125
TAS2R41R	TGAGGATCATATCCAAGGGCAG	
TAS2R42F	TGGACTGGTAAACTGCTCTGA	208
TAS2R42R	TGTCAAGTGATTAGTCATGTGCC	
TAS2R43F	TAATATCTGGGCAGTGATCAACC	150
TAS2R43R	CCCAACAACATCACCAGAATGA	

TAS2R45F	CGTGCCATTTACTTTGTGTCTGTA	122
TAS2R45R	ATGAACGGGTGGGCTGAAGAA	
TAS2R46F	GCAATGTACCTTTCAAATACAACG	193
TAS2R46R	GGAAGGAGGTCACAGTTTGC	
TAS2R50F	GATCTCAGCACCAAGGTCCA	140
TAS2R50R	CTAACCATGACAACCGGGTCA	
TAS2R60F	TACCTTCACCCACCCTGTCT	191
TAS2R60R	TCCGTATGCTATCGCCAGTG	
TAS2R19F2	TGCAACTGTGTTTAATTCTGCTT	89
TAS2R19R2	CACATGCTGAAATGGTTCGT	
TAS2R2F	TCTGGCCTCTGTGAGCATTG*	224
TAS2R2R	TTGCATCCGATGAGTGTGCT*	
Hprt1f	CAGTCCCAGCGTCGTGATTA	167
Hprt1r	GGCCTCCCATCTCCTTCATG	
Tas2r102f	CTCCTGCTAATCTTCTCTTTGTG	71
Tas2r102r	GGGTCTCTGTGTCTTCTGG	
Tas2r103f	GGGTTCTTGGTATCATTATTGGAC	80
Tas2r103r	ACCATCCAGGAAATAGTAAGGAG	
Tas2r104f	GCAACACATCCTGGCTGAT	71
Tas2r104r	CCCCATATTGGCAAAAACAT	
Tas2r105f	AAGGCATCCTCCTTTCCATT	69
Tas2r105r	GTGCAATAAATGTGTTCCCTAAAA	
Tas2r107f	GGCATCCTCCTTTGTGTTGT	66
Tas2r107r	TGCAATATATGTGTCCCCTAAAAC	
Tas2r110f	CTTTCTCATGCTCATCTTCTCAC	76
Tas2r110r	GGCATCTCTAGGTGGTTTGG	
Tas2r114f	CGGCTGCCACTCACTTATC	84
Tas2r114r	CAGCACTTTAATAGTTGCAGTATCATT	
Tas2r115f	CCTTTGGTGTATCCTTGATAGCTT	71
Tas2r115r	CTGCATCTTCCTTACATGTTTCA	
Tas2r117f	CCCTGTGGACACATCACAAG	93
Tas2r117r	TCACAGTTTGTAGGGCTTTGAA	
Tas2r118f	CACTGGGTGCAGATGAAACA	69
Tas2r118r	CTTCAGAACAGTGAACTGAGCTTT	
Tas2r121f	CTGGTCTTATTGGAGATGATTGTG	81
Tas2r121r	GGAGAAGATTAACAGGATGAAGGA	
Tas2r123f	CATTAAAGCCTTGCAAACTGTG	62
Tas2r123r	GGAAAAGTAAGTATATGGCATACAGCA	
Tas2r124f	CTCCACCATCATACTAATTGCAG	80
Tas2r124r	AGTCAATGCAGTTCTTCAACAC	
Tas2r130f	TGCATTCATTGCACTGGTAAA	70
Tas2r130r	GATTAAATCAATAGAGGCAATCTTCC	
Tas2r106f2	GCACAGAAATGTTTCCTGGCA	453
Tas2r106r2	GTCACTCTGACGTCCTTGTCT	

Tas2r126f2	TCCTCTTCAGTTTGGGCACC	285
Tas2r126r2	CGGACACCAAGATAGAGCCC	
Tas2r135f2	GAGTGGCCATCAACCTTGGA	288
Tas2r135r2	GCAGAACTGAGTACCAGCGT	
Tas2r138f2	AGCTTTCCTGGTTTCCTCGG	366
Tas2r138r2	GGAGGAACCTTGTGGACTGG	
Tas2r109f2	GCATGGACTGGGTTAAGAGAAAA	113
Tas2r109r2	GCTAATTGGAAAACCTACTAGCATGA	
Tas2r116f2	TCCATTTGCCAACACCATGT	77
Tas2r116r2	GGGAGAAAACAAGAAGGACAAAAG	
Tas2r119f2	CAAGAGCTTGGGTCACCTCAA	75
Tas2r119r2	TGTTGGCTGAGTGATGAGTAGCA	
Tas2r120f2	CTTGTGATTTTCCTGGGATCGT	80
Tas2r120r2	CTCATCCAAGCAATCTTCTTGATG	
Tas2r125f2	TTGGCTTTTCTGCTTCTCATCTT	79
Tas2r125r2	TGTCTTCGGAGCCTTTAGCATAG	
Tas2r129f2	TGCAACAGCATGCACAAAGA	72
Tas2r129r2	TGACCATGGTTTGCAAGACTCT	
Tas2r137f3	GTCTCAGCATCACTCGGCTTT	78
Tas2r137r3	GCAGGCGAGCTGAATAGCA	
Tas2r108f3	ACTTGGGTCAACAGTCGCAG	71
Tas2r108r3	AGGAATCTAGTGATGGCCAAGC	
Tas2r143f3	AGTGTCCGTTATCGTGCTCA	220
Tas2r143r3	GATAGCCTCTCTGGCCCAAT	
Tas2r122f2	TGGTTCAAGAATCAGAAAATCTCTGT	91
Tas2r122r2	CAGCAATTGTTGTCCATAGAACACA	
Tas2r131f2	CTGCCTGAGCATATTCTACTTATTCAA	83
Tas2r131r2	CACCTCTCAATCTCCACTTAAACCA	
Tas2r134f2	GGCAGGGAATGGGTACGAA	73
Tas2r134r2	ACCTGGAGGCAGCTAAGCAA	
Tas2r136f2	CAATGAGGCTTTATGGAAAAGGA	77
Tas2r136r2	TGAGATCAAAACTTGCAAAACCTTA	
Tas2r139f2	ACACACCCTGAACATGAGAAACA	70
Tas2r139r2	GGCCTGCATATGAGCCTCTATG	
Tas2r113f2	AAGAATATGCAGCACACCGC	74
Tas2r113r2	ATGGTTTGCAGGGCTCTGAT	
Tas2r140f2	CACTTGCTTTTCCTTCGGGC	99
Tas2r140r2	AACTGGACTTCAGCCACCAT	
Tas2r144f3	CTCACTCAAGAGGCACACCC	93
Tas2r144r3	GTCGATTTGATGGCACCCAC	
Tas2r109f3	ACTGGTGTCTCTGGTCCTCTT	298
Tas2r109r3	TTGCAAGGCTTTGAAGTGGG	
Tas2r130f2	GCTGTTGGTGAGGCCTTAGT	510
Tas2r130r2	GACAGAGGCATGTCCAGCTT	

Tas2r144f5	CACGTGGGTGCCATCAAATC	221
Tas2r144r5	TGAACATGGTGCTGAAACCG	
Tas2r144f6	ATGGCAATAATTACCACAAATTCTGACT**	960
Tas2r144r6	CTACCTTTTAAGGTAAAGATGAACATGG**	

*: Primers used to get the full length CDS of *Tas2r144*; **: Primers used for amplification and sequencing of *TAS2R2*.

Table S2 Sequences of siRNAs for TAS2R knockdown.

RNA name	Sequences	
Scramble-h	UUCUCCGAACGUGUCACGU	ACGUGACACGUUCGGAGAA
siTAS2R46-1	UAAACUGACAUGAUUAUGG	CCAUAAUCAUGUCAGUUUA
siTAS2R46-2	UUUGUCUCUUGAACCACUC	GAGUGGUUCAAGAGACAAA
siTAS2R46-3	AACAACACUCUUAACUCUC	GAGAGUUAAGAGUGUUGUU
Scramble-m	UUCUCCGACAGUGUCACGU	ACGUGACACUGUCGGAGAA
siTas2r135-1	GAGCUUAACUAGUAUCCUA	UAGGAUACUAGUUAAGCUC
siTas2r135-2	CCUGUAAUACAGCUCCUAA	UUAGGAGCUGUAUUACAGG
siTas2r135-3	GAAUAGCUUAAGACACUCA	UGAGUGUCUUAAGCUAUUC
